# Nitrogen-Containing Secondary Metabolites from a Deep-Sea Fungus *Aspergillus unguis* and Their Anti-Inflammatory Activity

**DOI:** 10.3390/md20030217

**Published:** 2022-03-20

**Authors:** Cao Van Anh, Yeo Dae Yoon, Jong Soon Kang, Hwa-Sun Lee, Chang-Su Heo, Hee Jae Shin

**Affiliations:** 1 Marine Natural Products Chemistry Laboratory, Korea Institute of Ocean Science and Technology, 385 Haeyang-ro, Yeongdo-gu, Busan 49111, Korea; caovananh@kiost.ac.kr (C.V.A.); hwasunlee@kiost.ac.kr (H.-S.L.); science30@kiost.ac.kr (C.-S.H.); 2 Department of Marine Biotechnology, University of Science and Technology (UST), 217 Gajungro, Yuseong-gu, Daejeon 34113, Korea; 3 Laboratory Animal Resource Center, Korea Research Institute of Bioscience and Biotechnology, 30 Yeongudanjiro, Cheongju 28116, Korea; yunyd76@kribb.re.kr (Y.D.Y.); kanjon@kribb.re.kr (J.S.K.)

**Keywords:** deep-sea fungus, *A. unguis*, variotin, coniosulfide, anti-inflammatory

## Abstract

*Aspergillus* is well-known as the second-largest contributor of fungal natural products. Based on NMR guided isolation, three nitrogen-containing secondary metabolites, including two new compounds, variotin B (**1**) and coniosulfide E (**2**), together with a known compound, unguisin A (**3**), were isolated from the ethyl acetate (EtOAc) extract of the deep-sea fungus *Aspergillus unguis* IV17-109. The planar structures of **1** and **2** were elucidated by an extensive analysis of their spectroscopic data (HRESIMS, 1D and 2D NMR). The absolute configuration of **2** was determined by comparison of its optical rotation value with those of the synthesized analogs. Compound **2** is a rare, naturally occurring substance with an unusual cysteinol moiety. Furthermore, **1** showed moderate anti-inflammatory activity with an IC_50_ value of 20.0 µM. These results revealed that *Aspergillus unguis* could produce structurally diverse nitrogenous secondary metabolites, which can be used for further studies to find anti-inflammatory leads.

## 1. Introduction

Deep-sea hydrothermal vents are recognized as one of the most extreme and dynamic habitats on our planet [1]. These hotspot ecosystems are characterized by high temperature, high pressure, low oxygen supply, and the absence of sun light [2]. In addition, hydrothermal vent flows bring fluids with high concentrations of reduced sulfur-containing compounds and heavy metals [2]. Given this fact, microorganisms living in this specific environment are considered as a new frontier for discovery of natural products with unique structures and tremendous pharmacological activities [3].

*Aspergillus* is renowned as a prolific source of numerous fungal peptides, including lipo-, depsi-, linear-, and cyclic-peptides, which are structurally unique and demonstrated various bioactivities, such as anti-microbial, anti-fungal, anti-inflammatory, and cytotoxic activities [4,5]. Among the peptides derived from *Aspergillus* spp., unguisins are a unique cyclic heptapetide class commonly produced by *Aspergillus unguis*, and until now unguisins A–G have been reported [6,7].

Inflammation is a protective response of our body to a wide range of stimuli. This process plays a central role or is an important symptom in the pathogenesis of various chronic diseases for instance Alzheimer’s disease, asthma, diabetes, and rheumatoid arthritis [8]. The inflammatory process is characterized by over secretion of nitric oxide (NO) and inflammatory cytokines such as interleukin 1 beta (IL-1β), tumor necrosis factor alpha (TNF-α), and interleukin 6 (IL-6). Therefore, reducing the production of inflammatory mediators is a key indicator for the treatment of various diseases. 

**Figure 1:**
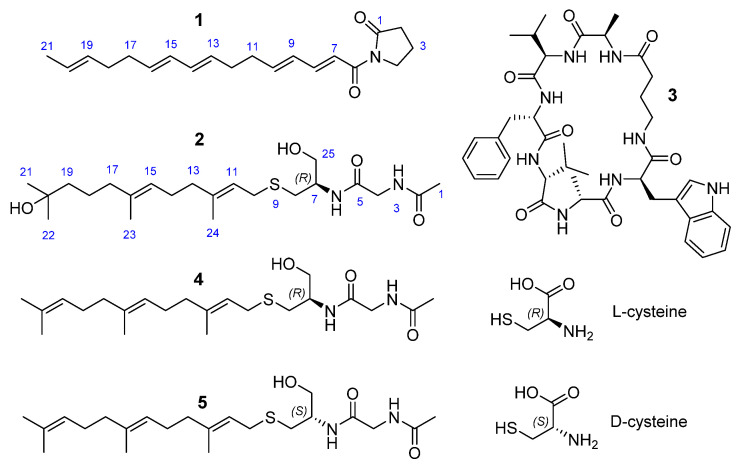
Structures of **1**–**3** isolated from *A. unguis* IV17-109, and the synthetic analogs (**4** and **5**).

As part of our study on marine-derived microorganisms isolated near hydrothermal vents, we have reported some anti-inflammatory phenazine alkaloids from a yeast-like fungus *Cystobasidium laryngis*, and nidulin-related polyketides from *A. unguis* IV17-109, which showed anti-microbial and cytotoxic activities [9,10]. Based on NMR guided isolation, we found that the ^1^H NMR spectra of non-polar fractions from *A. unguis* IV17-109 showed some minor interesting peaks in the olefinic region, which do not belong to unguisin peptides or nidulin-related polyketides. Further careful purification of these fractions led to the identification of two new compounds, variotin B (**1**) and coniosulfide E (**2**) (Figure 1). Anti-inflammatory activity of **1** and **2** was preliminarily evaluated and the result revealed that **1** has moderate activity. Here, we report the details of the isolation, structure identification, and anti-inflammatory nature of these compounds.

## 2. Results and Discussion

**Figure 2:**
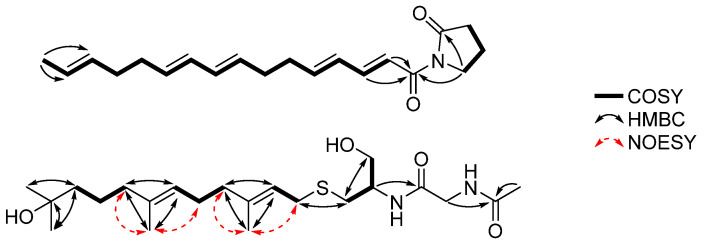
Key 2D NMR data of **1** and **2**.

Compound **1** was isolated as pale-yellow needles with the molecular formula of C_20_H_27_NO_2_ based on its HRESIMS peak at *m*/*z* 336.1938, ([M+Na]^+^, calculated for C_20_H_27_NO_2_Na, 336.1939), requiring 8 indices of hydrogen deficiency. The ^1^H NMR spectrum of **1** showed signals attributed to a methyl group at *δ*_H_ 1.62 (d, *J* = 4.5, H_3_-21), seven methylene groups at *δ*_H_ 2.04–3.82, and ten olefinic protons at *δ*_H_ 5.42–7.37. The ^13^C NMR spectrum in combination with HSQC data revealed signals of 20 resonances belonging to a methyl at *δ*_C_ 18.1, seven methylene carbons at *δ*_C_ 18.1–47.0, ten olefinic carbons at *δ*_C_ 121.7–146.9, and two carbonyl carbons at *δ*_C_ 168.2 and 177.8. Two carbonyl and ten sp^2^ carbons, accounting for 7 out of 8 degrees of unsaturation, indicated **1** is a monocyclic compound. The structure of a five-membered lactam ring was determined by continuous ^1^H-^1^H COSY correlations from H_2_-2 to H_2_-4, and the HMBC correlation from H_2_-4 to C-1. A substructure was identified as a C-16 polyunsaturated fatty acid by continuous ^1^H-^1^H COSY correlations from H-7 to H_3_-21, and the HMBC correlations from H-7 and H-8 to C-6 (Figure 2). The connection of the fatty acid and the lactam ring was corroborated by the HMBC correlation from H_2_-4 to C-6. 

The geometries of *∆*^7,9,13,15^ were deduced as *E*-form by their large coupling constants (Table 1) and the chemical shift of terminal methyl (C-21) was *δ*_C_ 18.1, revealing the geometry of *∆*^19^ was *E*-form [11,12]. Therefore, **1** was determined as a new variotin derivative with a non-branched side chain and named variotin B [13]. 

Compound **2** was isolated as a colorless solid and its molecular formula was determined as C_22_H_40_N_2_O_4_S, with four indices of hydrogen deficiency based on its HRESIMS peak at *m*/*z* 451.2607, ([M+Na]^+^, calculated for C_22_H_40_N_2_O_4_SNa, 451.2606). The ^1^H NMR spectrum of **2** showed signals attributed to five methyl groups at *δ*_H_ 1.17 (s, 6H, H_3_-21 and H_3_-22), 1.61 (s, H_3_-23), 1.68 (s, H_3_-24), and 2.01 (s, H_3_-1); seven methylene groups at *δ*_H_ 1.40–3.22; an oxygenated methylene group at *δ*_H_ 3.60 and 3.64 (H-25_a,b_); an amide methylene at *δ*_H_ 3.86 (m, H_2_-4); an amide methine at *δ*_H_ 4.03 (m, H-7); and two olefinic protons at *δ*_H_ 5.13 and 5.23 (m, H-11 and H-15). The ^13^C NMR spectrum in combination with HSQC data demonstrated signals of 22 resonances belonging to five methyls at *δ*_C_ 16.0 (C-23), 16.2 (C-24), 22.5 (C-1), and 29.2 (2C, C-21 and C-22); seven methylenes at *δ*_C_ 23.7–44.3; an oxygenated methylene at *δ*_C_ 63.6; an amide methylene at *δ*_C_ 43.6; an amide methine at *δ*_C_ 52.3; a tertiary alcohol at *δ*_C_ 71.4; four olefinic carbons at *δ*_C_ 121.8–140.1; and two carbonyl carbons at *δ*_C_ 171.5 and 173.9. Two carbonyl and four sp^2^ carbons accounting for all 4 degrees of unsaturation indicated **2** is an acyclic compound. 

The structure of a cysteinol unit was determined by sequential ^1^H-^1^H COSY correlations of H-8_a,b_/H-7/H-25_a,b_. A partial structure of *N*-acetylglycine, which was connected to the cysteinol moiety via a peptide bond, was determined by the HMBC correlations of H-7/C-5, H_2_-4/C-2, and H_3_-1/C-2. The remaining 15 carbons were assigned as a 10-hydro-11-hydroxyfarnesyl moiety based on a detailed analysis of ^1^H-^1^H COSY and HMBC data (Figure 2), and the connection of this moiety with the cysteinol residue via a thioether bond was determined by the HMBC correlations of H-8 _a,b_/C-10 and H-10_a,b_/C-8. The geometry of *∆*^11^ was deduced as *E*-form by the strong NOESY correlations from H_3_-24 to H-10_a,b_ and H-13_a,b_; and no-observed NOESY correlation from H_3_-24 to H-11. Similarly, *∆*^15^ was also determined as *E*-form (Figure 2). Consequently, the gross structure of **2** was determined as shown in Figure 1. 

**Scheme 1:**
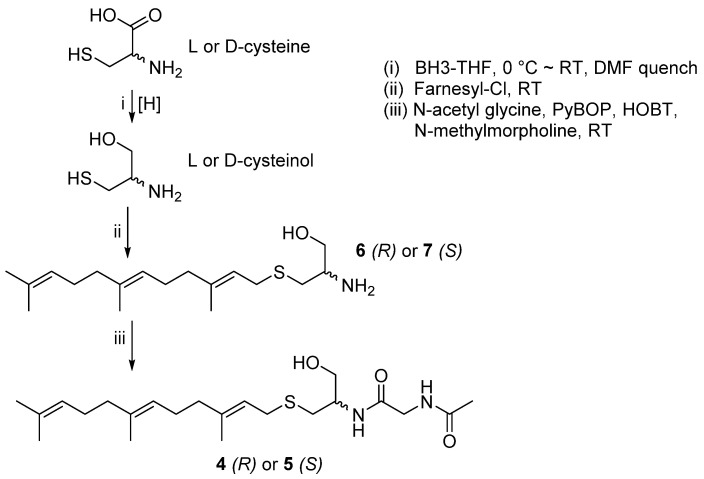
Synthesis of **4** and **5**.

To determine the absolute configuration of **2**, we synthesized its analogs (**4** and **5**, a pair of enantiomers synthesized from *L*- and *D*-cysteine and farnesyl chloride, Scheme 1) from commercially available substances. By comparing the optical rotation sign of **2** ${\left[\alpha \right]}_{D}^{20}$ − 100 (*c* 0.3, MeOH) with that of **4** ${\left[\alpha \right]}_{D}^{20}$ − 110 (*c* 0.3, MeOH) and **5** ${\left[\alpha \right]}_{D}^{20}$ + 120 (*c* 0.3, MeOH), the absolute configuration of **2** was determined to be the same as that of **4** (7*R*). Thus, **2** was determined as a new derivative of sulfur-containing natural products, coniosulfides A-D [14], and named coniosulfide E.

A co-isolated known compound was identified as unguisin A (**3**) by comparing its spectroscopic data with the corresponding literature values [6].

Since some fungal peptides were reported to show anti-inflammatory activity [4], **1** and **2** were evaluated for their anti-inflammatory activity. Subsequently, **1** showed moderate anti-inflammatory activity with an IC_50_ value of 20.0 µM. Even though a literature review revealed many synthetic analogs of **2** demonstrated inhibitory effects on human isoprenylcysteine carboxyl methyltransferase (hIcmt) [15] or the inflammation process [16], unfortunately, **2** showed no anti-inflammatory activity at a concentration of 30.0 µM. Due to the limited amount of **2**, we were unable to check its effect on hIcmt. Therefore, further studies are needed to find the bioactivities of **2**. 

**Figure 3:**
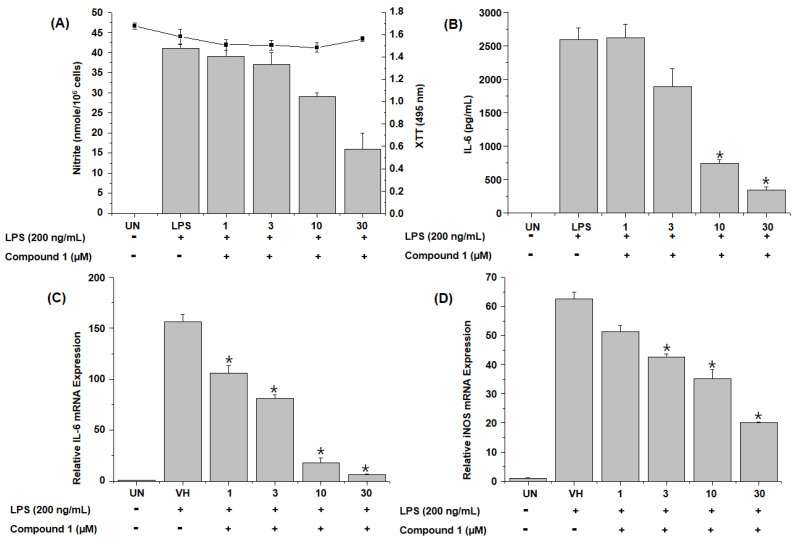
Inhibitory effects of **1** on LPS-induced nitrite production and IL-6 secretion in RAW 264.7 cells. RAW 264.7 cells were pretreated with **1** at the depicted concentrations (1~30 µM) for 1 h and stimulated with LPS (200 ng/mL) for 24 h. The levels of nitrite (**A**) and IL-6 (**B**) in culture supernatants were determined by Griess reaction and ELISA, respectively. The mRNA levels of IL-6 (**C**) and iNOS (**D**) were examined by qPCR. Data are represented as the mean ± SD of quadruplicate determinations. An asterisk (*) denotes that the response is significantly different from vehicle-treated group as determined by Dunnett’s multiple comparison test at *p* < 0.05. The results shown are representatives of more than two independent experiments (UN: Untreated; VH: Vehicle (0.1% DMSO)).

To further investigate the anti-inflammatory activity of **1**, we examined the inhibitory effect of **1** on lipopolysaccharide (LPS)-induced production of inflammatory mediators, including NO, IL-6, and iNOS, in RAW 264.7 cells. The treatment of RAW 264.7 cells with LPS led to the accumulation of nitrite and IL-6, and **1** dose-proportionally inhibited LPS-induced production of nitrite and IL-6 in LPS-stimulated RAW 264.7 cells (Figure 3A,B). To further examine whether the effect of **1** were due to its effects on the mRNA expression of cognate genes, we investigated the effect of **1** on the mRNA expression of inducible nitric oxide synthase (iNOS) and IL-6 by quantitative polymerase chain reaction (qPCR). The mRNA levels of iNOS and IL-6 were induced by LPS treatment, and this induction was suppressed by **1** in a concentration-dependent manner (Figure 3C,D). Considering the above-mentioned data, it is noticeable that **1** showed anti-inflammatory activity by suppressing the production of NO and the expression of iNOS and IL-6 with no cytotoxicity at the treated concentrations. The results revealed that fungal natural products could be an important source of leads for the development of new anti-inflammatory drugs with minimal side effects. 

## 3. Materials and Methods

### 3.1. General Experimental Procedures

HRESIMS data were obtained on a Waters Synapt G2 Q-TOF mass spectrometer (Waters Corporation, Milford, MA, USA). Optical rotations were measured on a Rudolph Research Analytical Autopol III polarimeter (Rudolph Research Analytical, Hackettstown, NJ, USA). 1D and 2D NMR spectra were acquired using a Bruker 600 MHz spectrometer (Bruker BioSpin GmbH, Rheinstetten, Germany). IR spectra were measured on a JASCO FT/IR-4100 spectrophotometer (JASCO Corporation, Tokyo, Japan). UV-visible spectra were measured by a Shimadzu UV-1650PC spectrophotometer. HPLC was carried out with a PrimeLine Binary pump (Analytical Scientific Instruments, Inc., El Sobrante, CA, USA) and a RI-101 detector (Shoko Scientific Co. Ltd., Yokohama, Japan). Semi-preparative HPLC was conducted using an ODS column (YMC-Pack-ODS-A, 250 × 10 mm i.d, 5 μM). Analytical HPLC was performed with an ODS column (YMC-Pack-ODS-A, 250 × 4.6 mm i.d, 5 μM). All the used reagents were purchased from Sigma-Aldrich (Merck KGaA, Darmstadt, Germany).

### 3.2. Fungal Material, Fermentation, and Isolation of Secondary Metabolites

#### Fungal Material, Fermentation, and Isolation of **1**–**3** from *Aspergillus unguis* IV17-109

*A. unguis* IV17-109 (GenBank accession number OL700797) was isolated from a deep-sea shrimp sample as previously described [10]. The EtOAc extract was fractionated into 10 fractions (F1–F10), as described earlier [10]. The F7 fraction was purified by a semi-preparative reversed-phase HPLC (YMC-Pack-ODS-A, 250 × 10 mm i.d, 5 µm, flow rate 2.0 mL/min, 60% MeOH/H_2_O, RI detector) to obtain **3** (2.0 mg, *t_R_* = 32 min). The F8 fraction was subjected to a semi-preparative reversed-phase HPLC (YMC-Pack-ODS-A, 250 × 10 mm i.d, 5 µm, flow rate 2.0 mL/min, RI detector) using an isocratic elution with 70% MeOH/H_2_O to yield a subfraction F8-1, and the subfraction was further purified by a semi-preparative HPLC (YMC-Pack-ODS-A, 250 × 10 mm i.d, 5 µm, flow rate 2.0 mL/min, RI detector) using an isocratic elution with 50% MeCN/H_2_O to obtain **2** (1.0 mg, *t_R_* = 15 min). Finally, the F9 fraction was purified by a semi-preparative reversed-phase HPLC (YMC-Pack-ODS-A, 250 × 10 mm i.d, 5 µm, flow rate 2.0 mL/min, RI detector) using an isocratic elution with 83% MeOH/H_2_O to yield **1** (3.0 mg, *t_R_* = 42 min).

Variotin B (**1**): pale-yellow needles; IR ν_max_ 3286, 2918, 1724, 1671, 1352, 1261, 989 cm^−1^; UV(MeOH) λ_max_ (log ε) 283 (2.51), 229 (2.60) nm; HRESIMS *m*/*z* 336.1938 [M+Na]^+^ (calcd for C_20_H_27_NO_2_Na, 336.1939), ^1^H NMR (CD_3_OD, 600 MHz) and ^13^C NMR (CD_3_OD, 150 MHz) see Table 1.

Coniosulfide E (**2**): colorless solid, ${\left[\alpha \right]}_{D}^{20}$ − 100 (c 0.3, MeOH); IR ν_max_ 3303, 2929, 1653, 1547, 1374, 1038 cm^−1^, HRESIMS *m*/*z* 451.2607 [M+Na]^+^ (calcd for C_22_H_40_N_2_O_4_SNa, 451.2606), ^1^H NMR (CD_3_OD, 600 MHz) and ^13^C NMR (CD_3_OD, 150 MHz) see Table 1.

### 3.3. Synthesis of **4** and **5**

Compounds **4** and **5** were synthesized according to the reported procedures with minor modifications [17]. Borane-tetrahydrofuran (Borane-THF, 4 mL, 4 mmol) was added dropwise to *L*- or *D*-cysteine (0.121 g, 1 mmol) in dry THF (5 mL) at 0°C under a nitrogen atmosphere, and stirred at ambient temperature for 7 h. The reaction mixture was quenched with dry dimethylfomamide (DMF, 1 mL) and stirred for 1 h. Farnesyl chloride (0.5 mmol) was added to the reaction mixture and stirred at room temperature for 3 h. The volatiles were removed in vacuo. The residue was re-dissolved in EtOAC (20 mL) and washed with H_2_O (20 mL) to remove the residue of cysteine. The EtOAc layer was evaporated under reduced pressure and the residue was purified by a semi-preparative HPLC using CH_3_OH/H_2_O (87:13) as an eluent to yield farnesyl-*L*-cysteinol (**6**) or farnesyl-*D*-cysteinol (**7**) (Figures S16 and S17).

To a dry DMF solution (500 μL) of **6** or **7** (5.0 mg) and *N*-acetylglycine (2.0 mg), benzotriazol-1-yloxytripyrrolidinophosphonium hexafluorophosphate (PyBOP, 12.0 mg), hydroxybenzotriazole (HOBT, 3.0 mg), and *N*-methylmorpholine (300 μL) were added [18]. The reaction mixture was stirred for 3 h at room temperature. Afterwards, 10 mL of water were added and the mixture was extracted twice with EtOAc (15 mL × 2). The EtOAC layer was dried, and the residue was purified by a semi-preparative HPLC (YMC-ODS column, 10 × 250 mm; MeCN-H_2_O, 65:35) to give compounds **4** or **5** with an overall yield of 11%.

1-farnesyl-2-(*N*-aetylglycine)-*L*-cysteinol (**4**): white amorphous solid, ${\left[\alpha \right]}_{D}^{20}$ − 110 (c 0.3, MeOH), ^1^H NMR (600 MHz, MeOD) *δ*_H_ 5.23 (t, *J* = 7.8 Hz, 1H), 5.14–5.07 (m, 2H), 4.05–4.00 (m, 1H), 3.90–3.82 (m, 2H), 3.64 (dd, *J* = 11.2, 5.2 Hz, 1H), 3.60 (dd, *J* = 11.1, 4.9 Hz, 1H), 3.22 (dd, *J* = 13.1, 8.0 Hz, 1H), 3.16 (dd, *J* = 13.1, 7.6 Hz, 1H), 2.70 (dd, *J* = 13.7, 6.5 Hz, 1H), 2.55 (dd, *J* = 13.7, 7.4 Hz, 1H), 2.11 (dt, *J* = 11.4, 5.8 Hz, 2H), 2.06 (dt, *J* = 14.2, 7.2 Hz, 4H), 2.01 (d, *J* = 1.0 Hz, 3H), 1.97 (t, *J* = 7.6 Hz, 2H), 1.68 (d, *J* = 7.2 Hz, 6H), 1.60 (s, 6H); ^13^C NMR (150 MHz, MeOD) *δ*_C_ 173.8, 171.4, 140.1, 136.2, 132.1, 125.4, 125.2, 121.8, 63.6, 52.3, 43.6, 40.8, 40.7, 32.9, 30.4, 27.8, 27.5, 25.9, 22.5, 17.8, 16.2, 16.1; ESIMS *m*/*z* 433.2 [M + Na]^+^ (Figures S18–S22).

1-farnesyl-2-(*N*-aetylglycine)-*D*-cysteinol (**5**): white amorphous solid, ${\left[\alpha \right]}_{D}^{20}$ + 120 (c 0.3, MeOH), ^1^H and ^13^C NMR, and ESIMS data of **5** were identical to those of **4** (Figures S23–S24).

### 3.4. Anti-Inflammatory Assay

Anti-inflammatory assay was conducted as described earlier [19]. Murine monocyte/macrophage RAW 264.7 (ATCC TIB-71) cell line was purchased from American Type Culture Collection (ATCC; Manassas, VA, USA).

## 4. Conclusions

In summary, based on NMR-guided isolation, two new (**1** and **2**) and one known (**3**) compounds were isolated from the culture broth of the deep-sea fungus *Aspergillus unguis* IV17-109. The planar structures of the new compounds were elucidated by a comprehensive analysis and comparison of their spectroscopic data with the values in the literature (HRESIMS, 1D, and 2D NMR). Compound **2** is a rare natural product with an unusual cysteinol moiety. The absolute configuration of **2** was determined by comparing its optical rotation sign with that of the synthesized analogs (**4** and **5**). Compounds **1** and **2** were preliminarily screened for their in vitro anti-inflammatory activity. Compound **1** showed moderate activity with an IC_50_ value of 20.0 µM. To the best of our knowledge, this is the first report on linear nitrogenous secondary metabolites isolated from *Aspergillus unguis*. This research expanded the biological and chemical diversities of fungal natural products.

## Figures and Tables

**Table 1:** ^1^H and ^13^C NMR data of **1** and **2** in CD_3_OD (600 MHz for ^1^H and 150 MHz for ^13^C).

Compound	1		2	
Position	*δ*_H_, mult (*J* in Hz)	*δ*_C_	*δ*_H_, mult (*J* in Hz)	*δ*_C_
1		177.8	2.01, s	22.5
2	2.61, t (8.1)	34.6		173.9
3	2.04, m	18.1		
4	3.82, m	47.0	3.86, m	43.6
5				171.5
6		168.2		
7	7.25, d (15.1)	121.7	4.03, m	52.3
8	7.37, dd (15.1, 10.8)	146.9	2.55–2.70	32.9
9	6.32, dd (15.1, 10.8)	130.7		
10	6.23, m	146.0	3.16–3.22	30.3
11	2.29, q (6.6)	34.0	5.23, td (7.8, 1.0)	121.8
12	2.21, m	32.8		140.1
13	5.56, td (13.6, 6.6)	131.6	2.06, m	40.7
14	6.01, m	132.6	2.13, m	27.4
15	6.01, m	131.8	5.13, td (6.8, 1.0)	125.2
16	5.56, td (13.6, 6.6)	133.1		136.3
17	2.10, dd (14.1, 6.8)	33.6	1.98, t (7.2)	41.3
18	2.04, m	33.8	1.46, m	23.7
19	5.44, m	131.9	1.40, m	44.3
20	5.44, m	126.1		71.4
21	1.62, d (4.5)	18.1	1.17, s	29.2
22			1.17, s	29.2
23			1.61, s	16.0
24			1.68, s	16.2
25			3.62, m	63.6

## Data Availability

The data presented in the article are available in the Supplementary Materials.

## Supplementary Materials

The following are available online at https://www.mdpi.com/article/10.3390/md20030217/s1, Figures S1–S15, HRESIMS data, ^1^H, ^13^C, ^1^H-^1^H COSY, HSQC, HMBC, NOESY NMR experimental spectra of compounds **1** and **2**. Figures S16–S24, ESIMS, ^1^H NMR, ^13^C NMR experimental spectra of **4**–**7**.
