# Supplementary material for: Nitrogen-Containing Secondary Metabolites from a Deep-Sea Fungus Aspergillus unguis and Their Anti-Inflammatory Activity
Source: Mar Drugs. 2022 Mar 20;20(3):217. doi: 10.3390/md20030217 (PMC8948696; doi:10.3390/md20030217)
Supplement: Supplementary file 1 [file marinedrugs-20-00217-s001.zip › marinedrugs-1641719-SI.pdf]

Nitrogen-Containing Secondary Metabolites from a Deep-Sea Fungus *Aspergillus unguis* and Their Anti-Inflammatory Activity

Cao Van Anh [1,2], Yeo Dae Yoon [3], Jong Soon Kang [3], Hwa-Sun Lee [1], Chang-Su Heo [1,2] and Hee Jae Shin [1,2,*]

[1] Marine Natural Products Chemistry Laboratory, Korea Institute of Ocean Science and Technology, 385 Haeyang-ro, Yeongdo-gu, Busan 49111, Korea; caovananh@kiost.ac.kr (C.V.A.); hwasunlee@kiost.ac.kr (H.-S.L.); science30@kiost.ac.kr (C.-S.H.)

[2] Department of Marine Biotechnology, University of Science and Technology (UST), 217 Gajungro, Yuseong-gu, Daejeon 34113, Korea

[3] Laboratory Animal Resource Center, Korea Research Institute of Bioscience and Biotechnology, 30 Yeongudanjiro, Cheongju 28116, Korea; yunyd76@kribb.re.kr (Y.D.Y.); kanjon@kribb.re.kr (J.S.K.)

* Correspondence: shinhj@kiost.ac.kr; Tel.: +82-51-664-3341; Fax: +82-51-664-3340

Contents

Figure S1. ^{1}H NMR spectrum of **1**. 3
Figure S2. ^{13}C NMR spectrum of **1**. 3
Figure S3. HSQC spectrum of **1**. 4
Figure S4. ^{1}H-^{1}H COSY spectrum of **1**. 5
Figure S5. HMBC spectrum of **1**. 6
Figure S6. NOESY spectrum of **1**. 7
Figure S7. HR-ESIMS data of **1**. 8
Figure S8. ^{1}H NMR spectrum of **2**. 9
Figure S9. ^{13}C NMR spectrum of **2**. 10
Figure S10. HSQC spectrum of **2**. 11
Figure S11. ^{1}H-^{1}H COSY spectrum of **2**. 12
Figure S12. HMBC spectrum of **2**. 13
Figure S13. 1D NOESY spectrum of **2**. 14
Figure S14. 1D NOESY spectrum of **2** (irradiated H_3-23) 14
Figure S14. 1D NOESY spectrum of **2** (irradiated H_3-24) 15
Figure S15. HR-ESIMS data of **2**. 16
Figure S16. ^{1}H and ^{13}C NMR spectra of **6**. 17
Figure S17. ^{1}H and ^{13}C NMR spectra of **7**. 18
Figure S18. ^{1}H and ^{13}C NMR spectra of **4**. 19
Figure S19. HSQC spectrum of **4**. 20
Figure S20. ^{1}H-^{1}H COSY spectrum of **4**. 21
Figure S21. HMBC spectrum of **4**. 22
Figure S22. LC-ESIMS data of **4**. 23
Figure S23. ^{1}H and ^{13}C NMR spectra of **5**. 24
Figure S24. LC-ESIMS data of **5**. 25

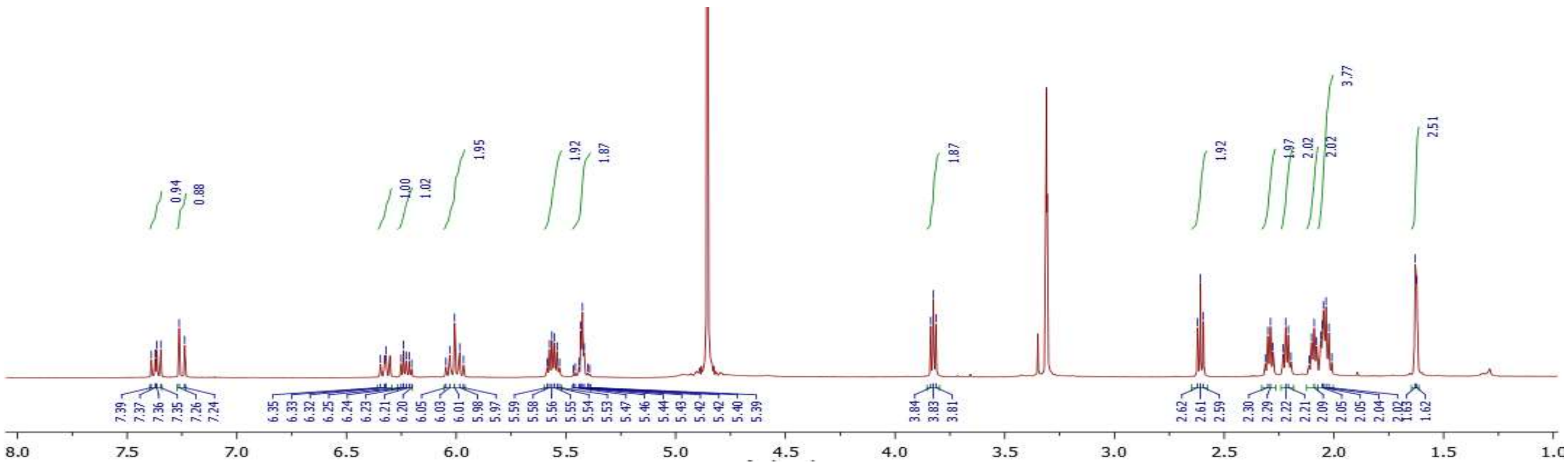

Figure S1. ^{1}H NMR spectrum of **1**.

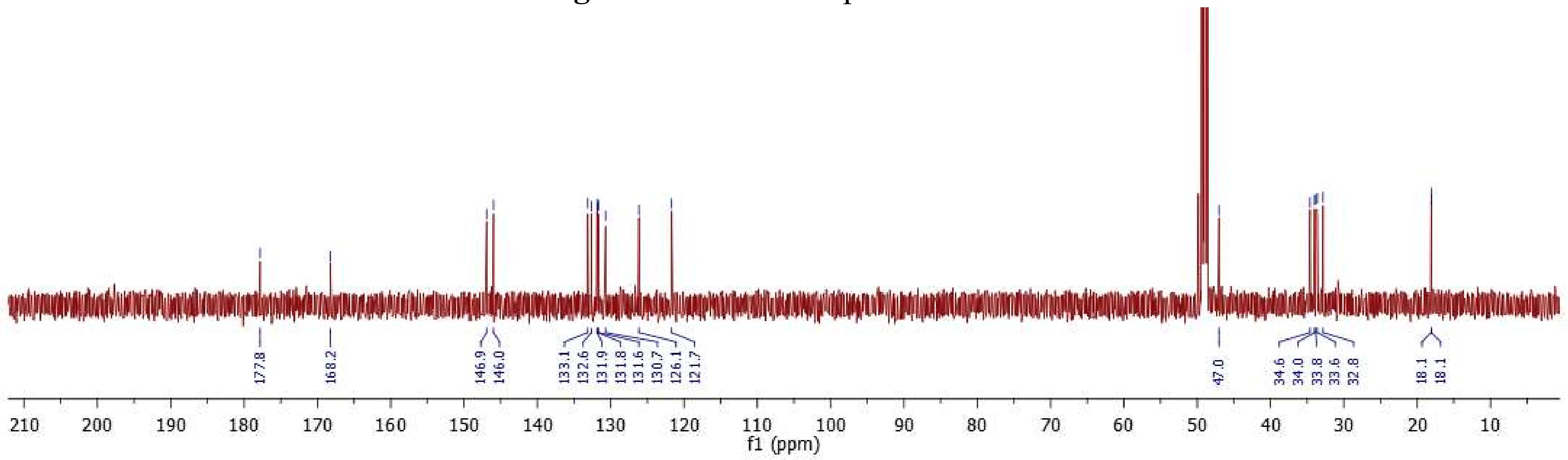

Figure S2. ^{13}C NMR spectrum of **1**.

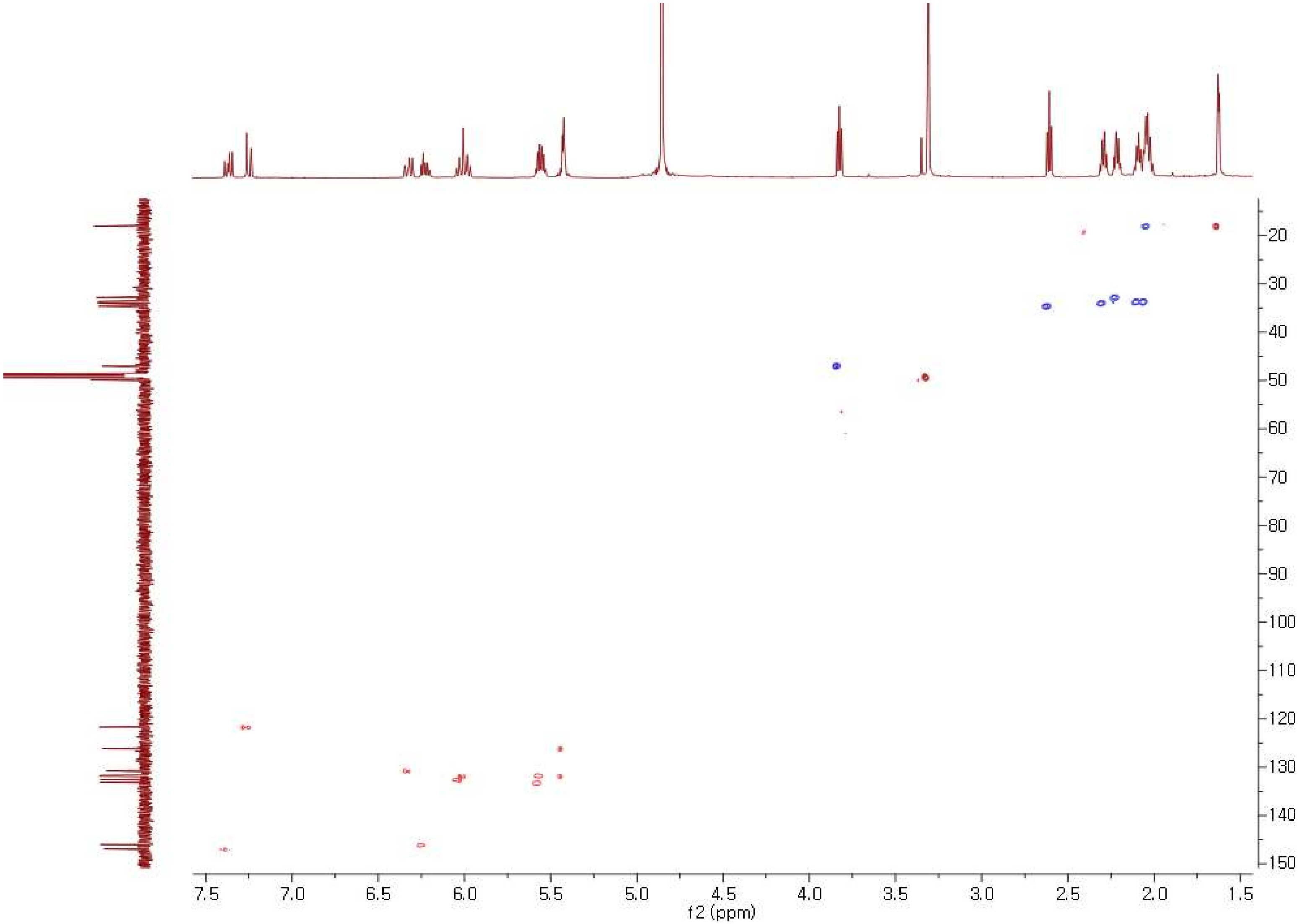

Figure S3. HSQC spectrum of **1**.

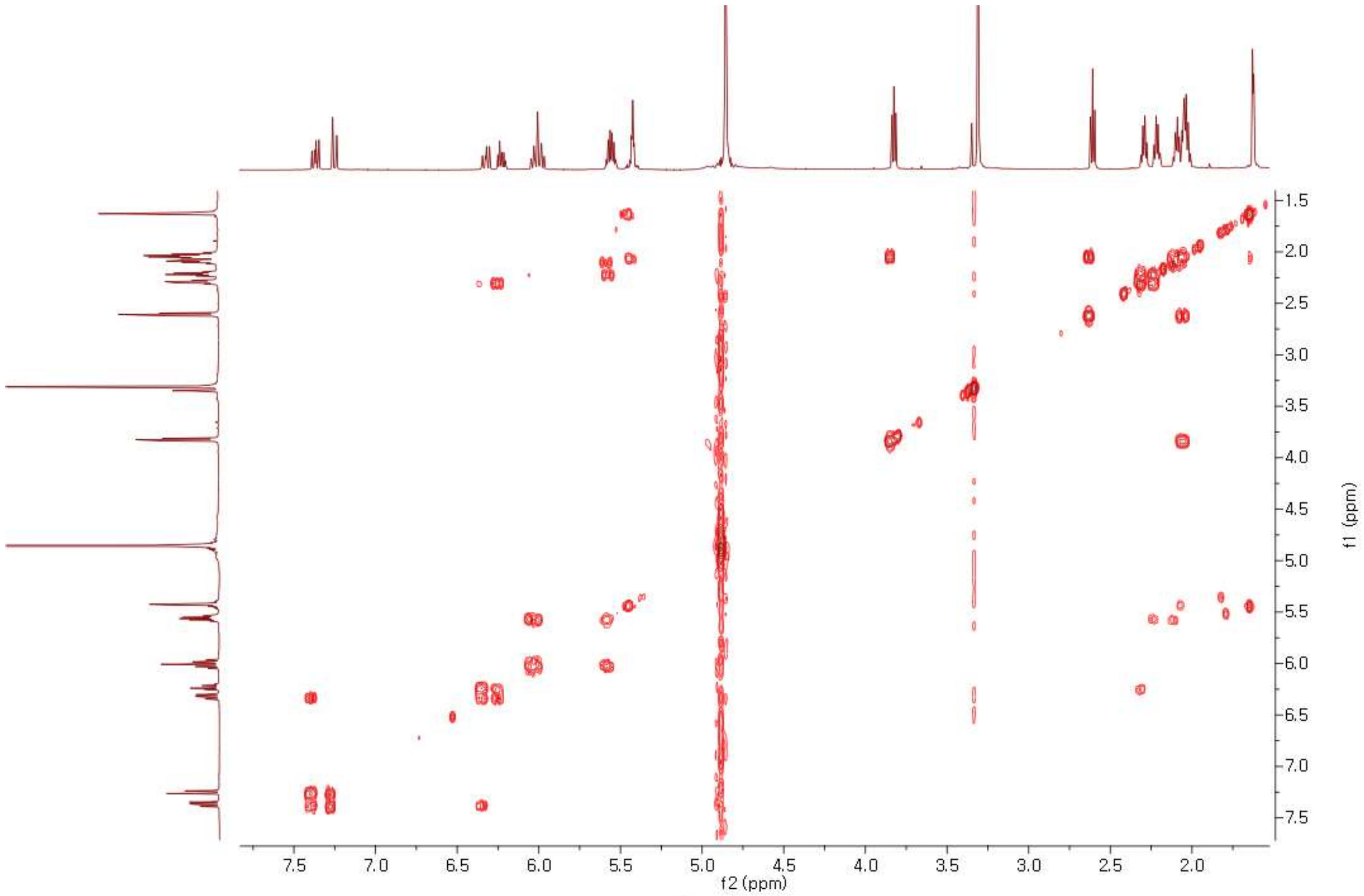

Figure S4. ^{1}H-^{1}H COSY spectrum of **1**.

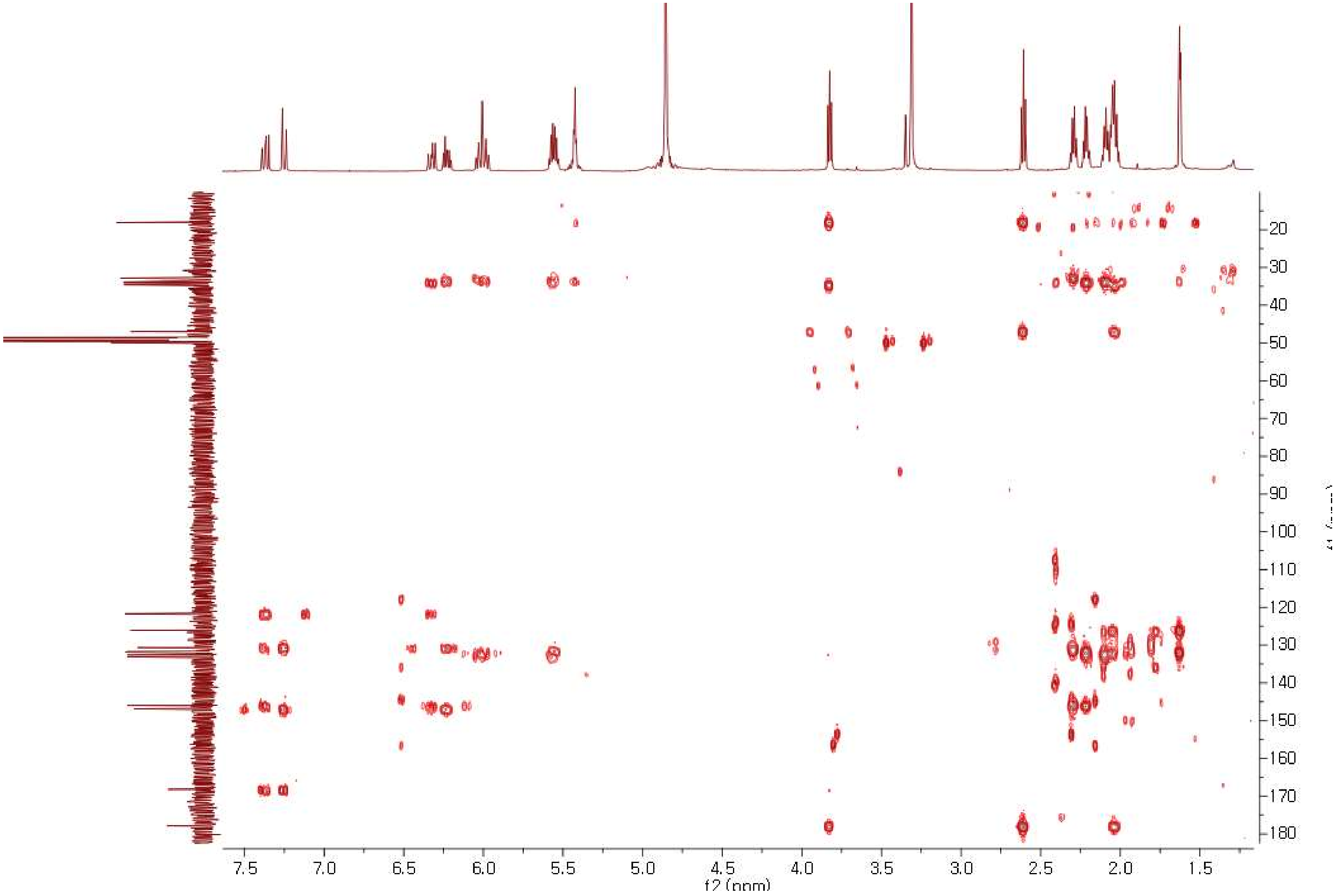

Figure S5. HMBC spectrum of **1**.

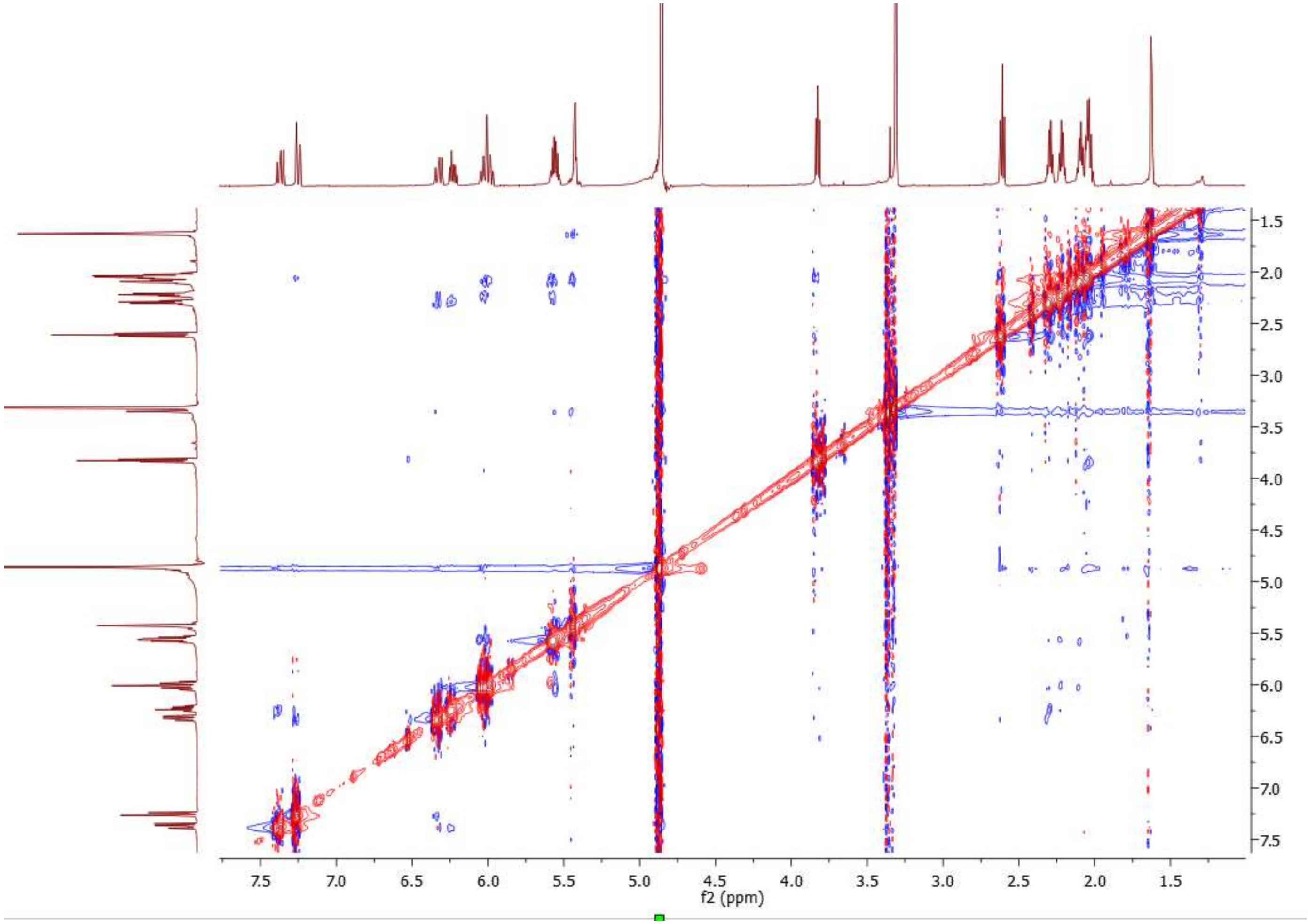

Figure S6. NOESY spectrum of **1**.

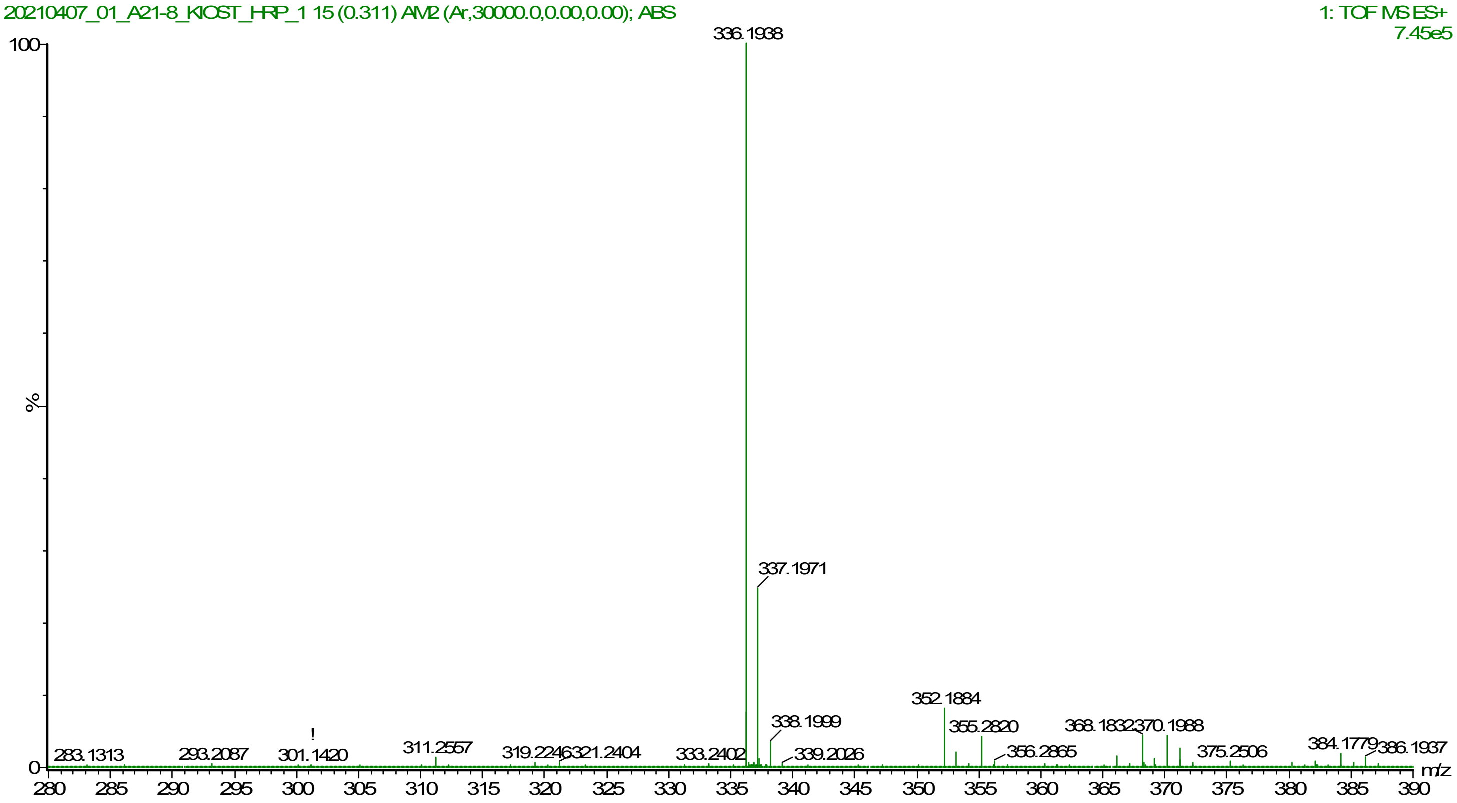

Elements Used:

C: 0-25 H: 0-30 N: 0-1 O: 0-2 Na: 0-1

Minimum:				-1.5				
Maximum:		100.0	5.0	100.0				
Mass	Calc. Mass	mDa	PPM	DBE	i-FIT	Norm	Conf(%)	Formula
336.1938	336.1939	-0.1	-0.3	7.5	1123.2	n/a	n/a	C20 H27 N O2 Na

Figure S7. HR-ESIMS data of **1**.

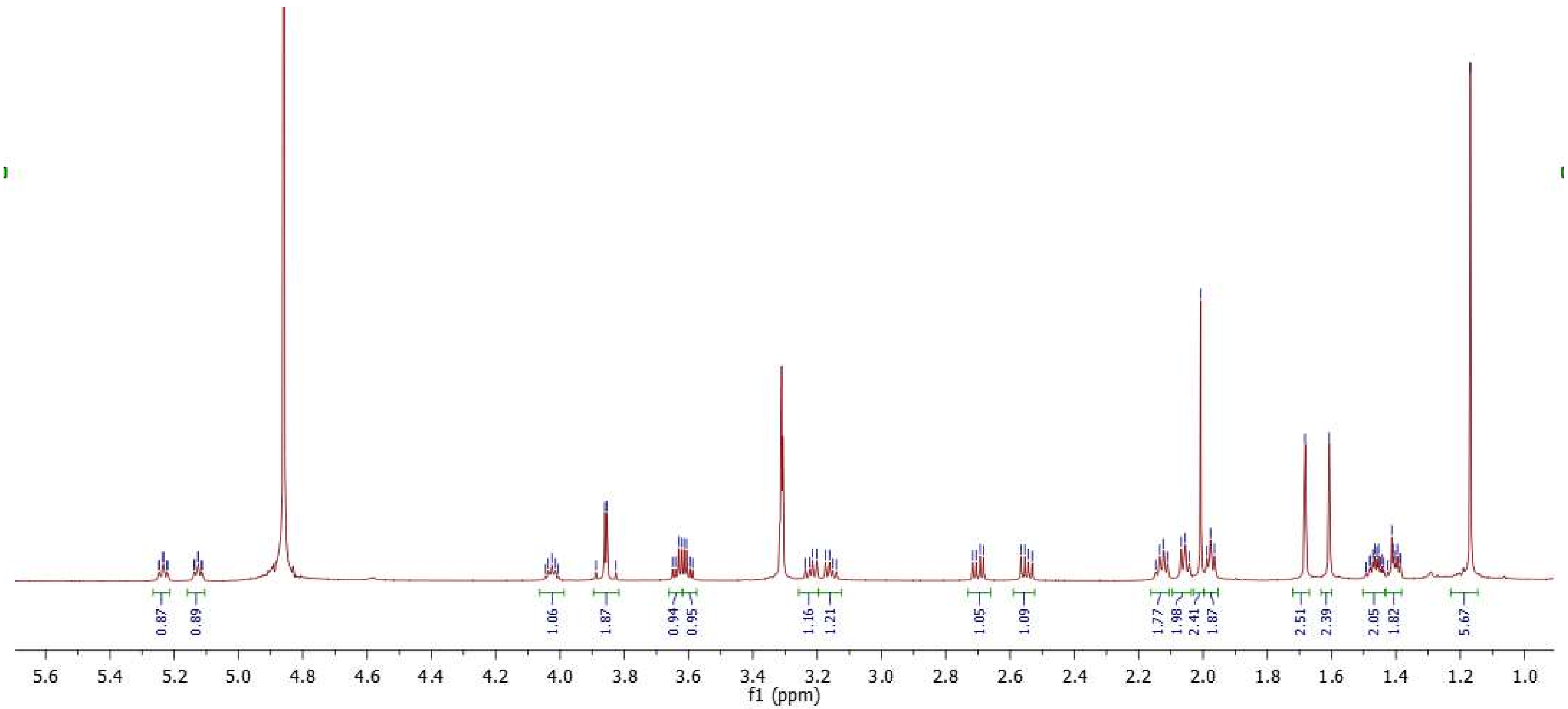

Figure S8. ^{1}H NMR spectrum of **2**.

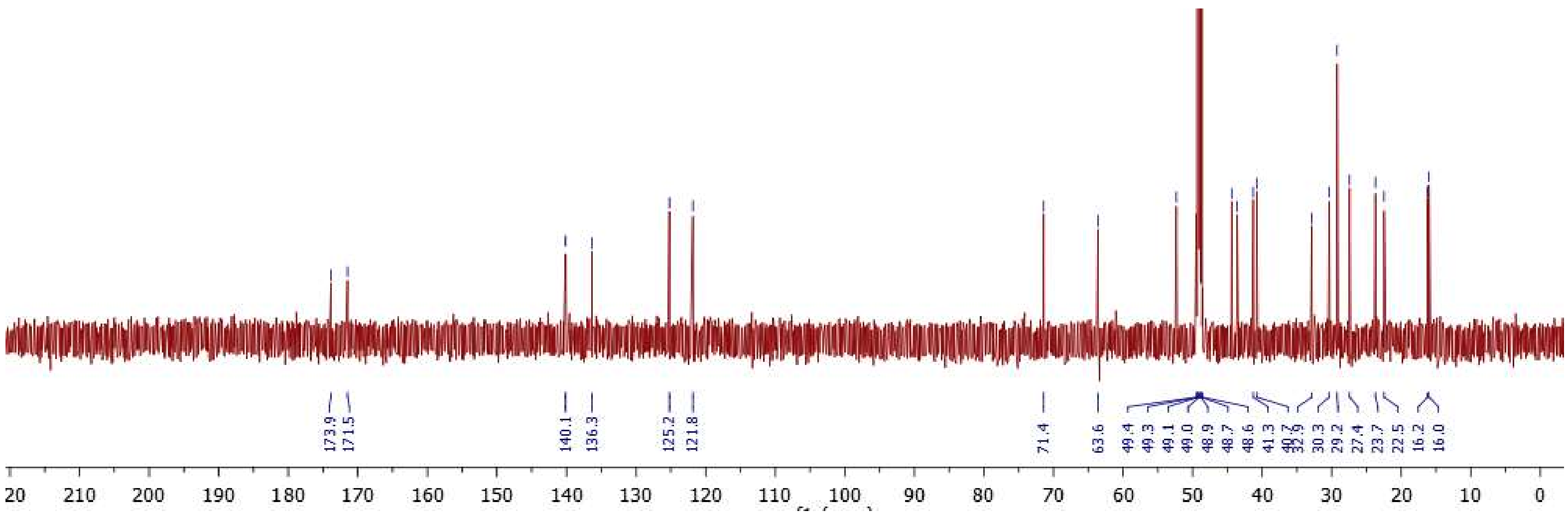

Figure S9. ^{13}C NMR spectrum of **2**.

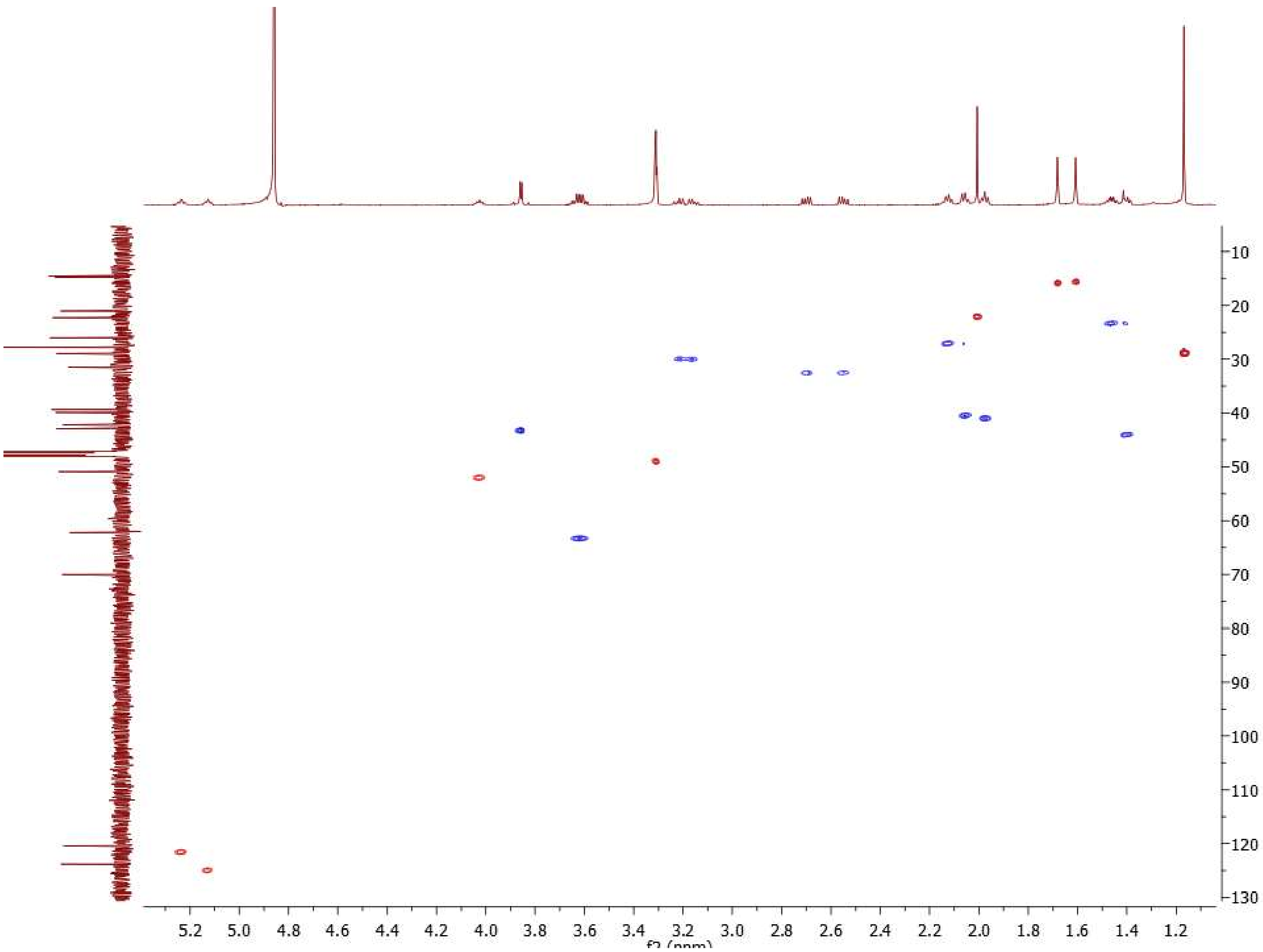

Figure S10. HSQC spectrum of **2**.

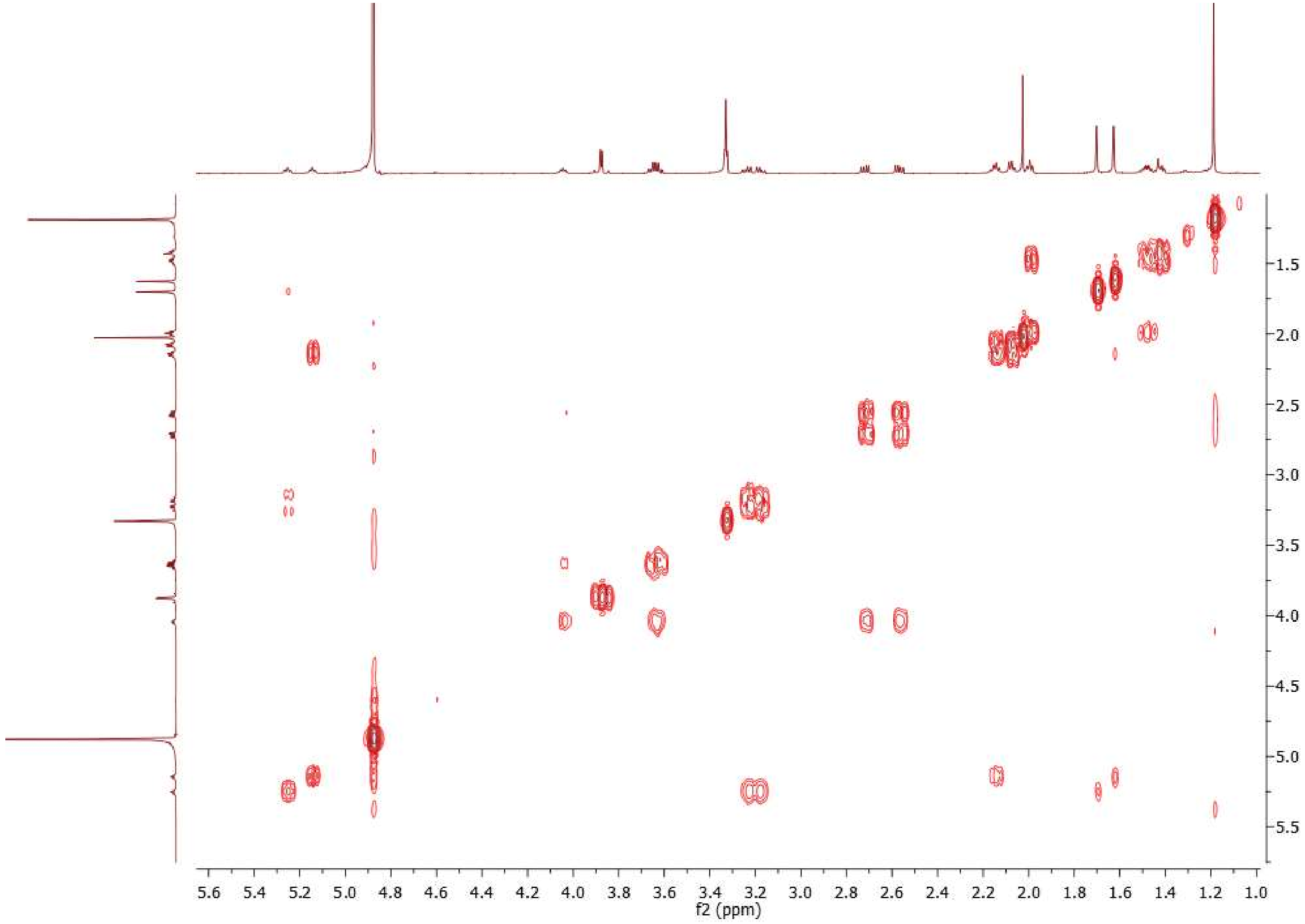

Figure S11. ^{1}H-^{1}H COSY spectrum of **2**.

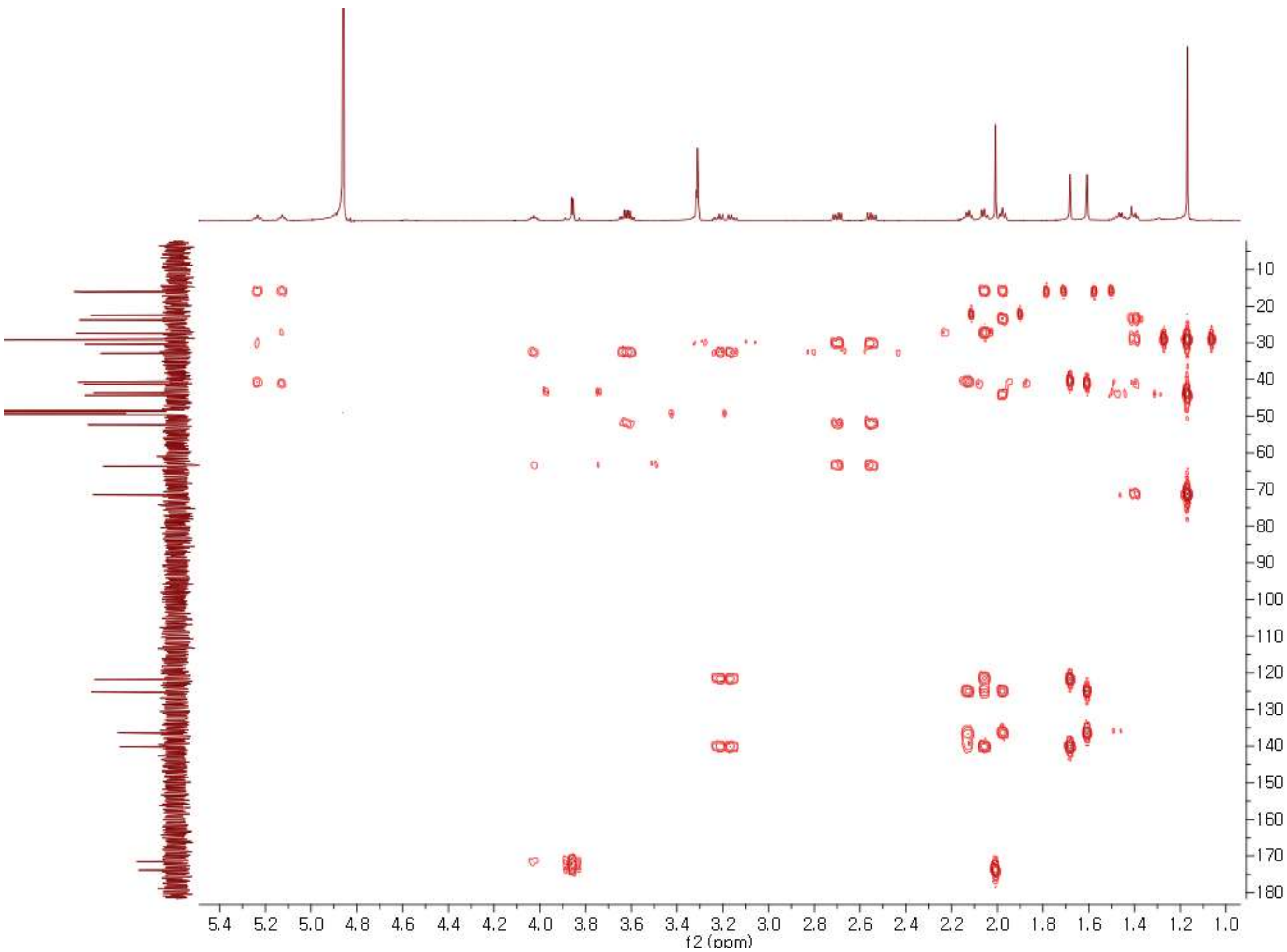

Figure S12. HMBC spectrum of **2**.

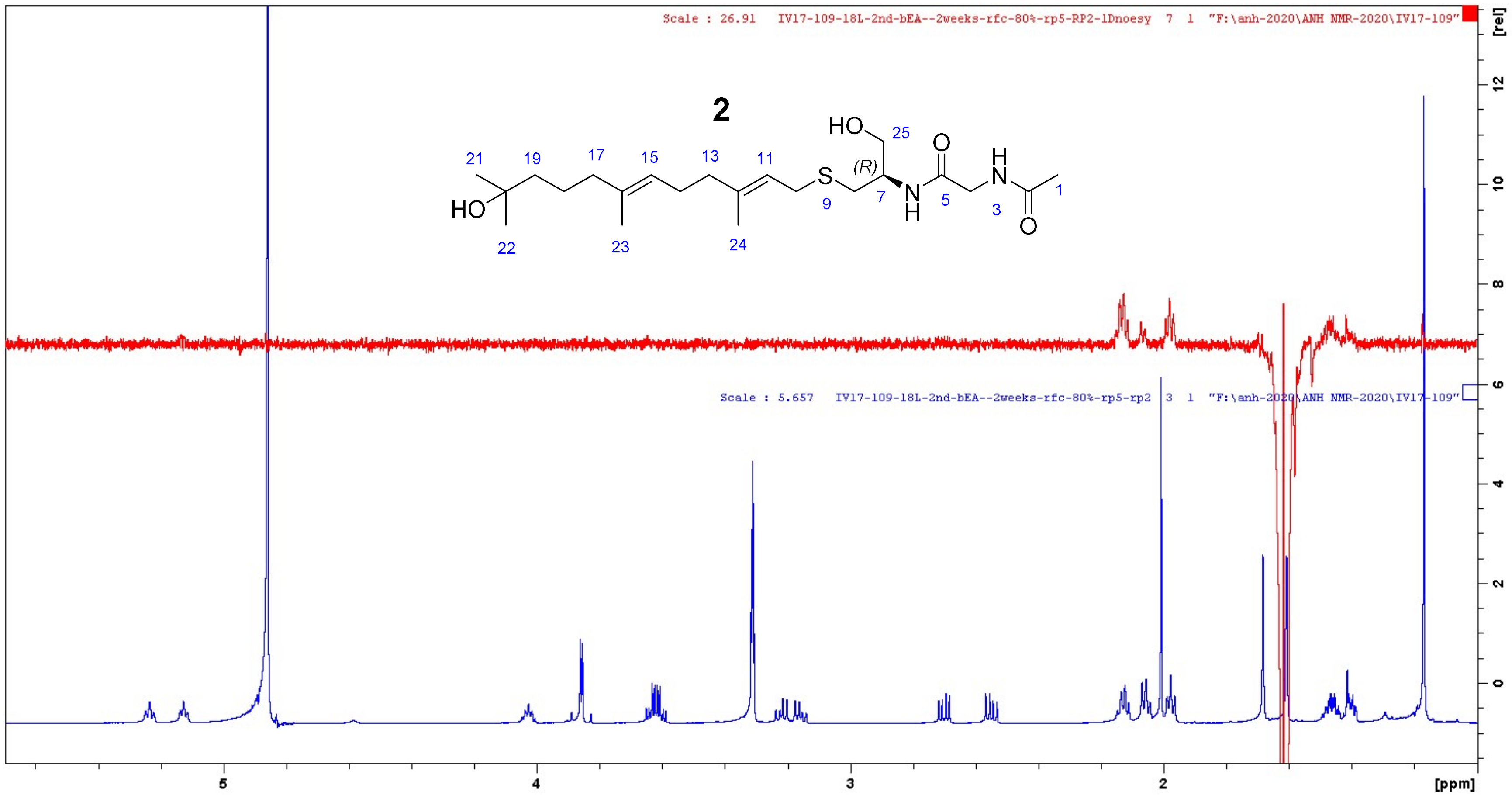

Figure S14. 1D NOESY spectrum of **2** (irradiated H_3-23).

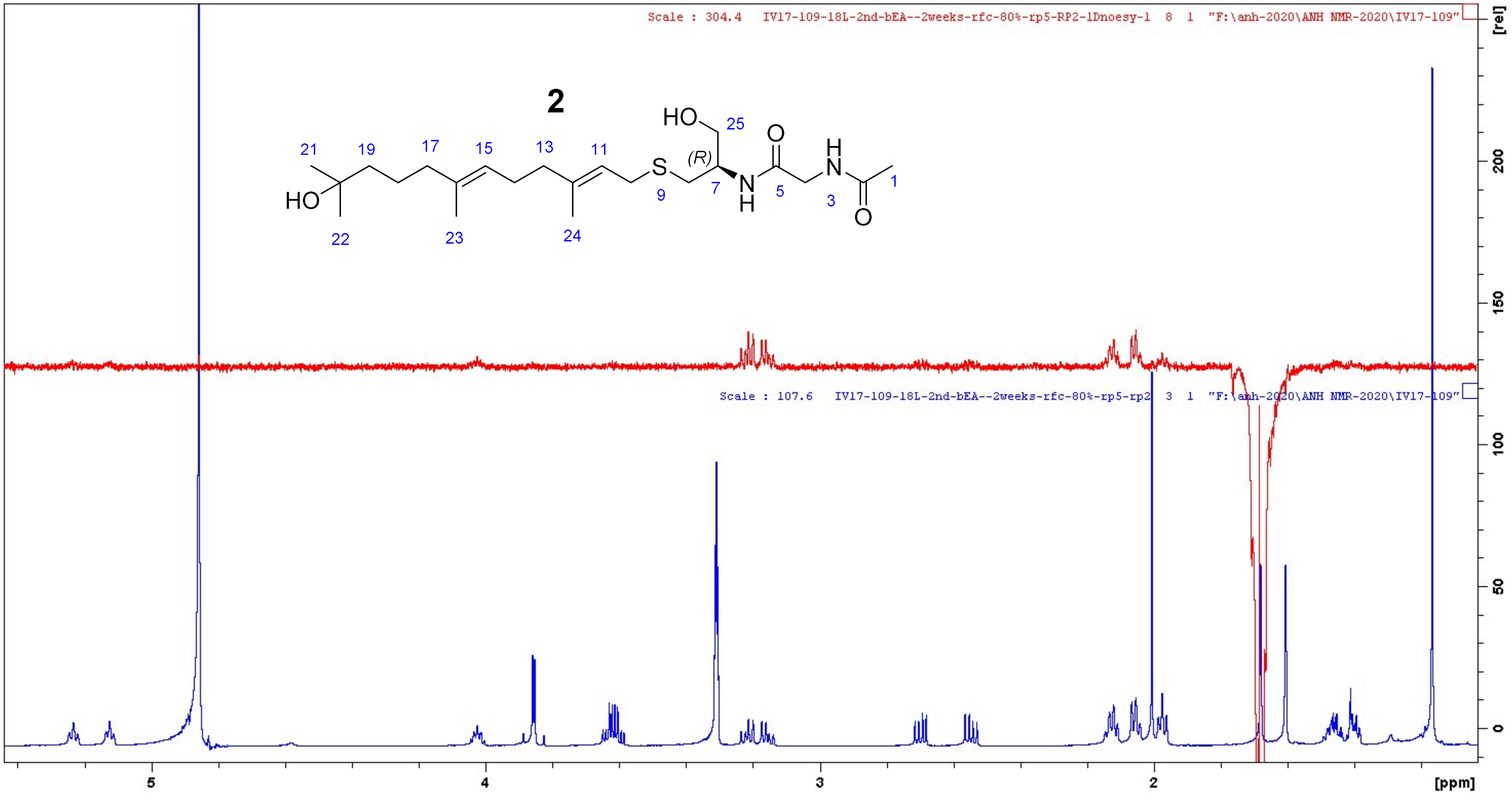

Figure S14. 1D NOESY spectrum of **2** (irradiated H_3-24).

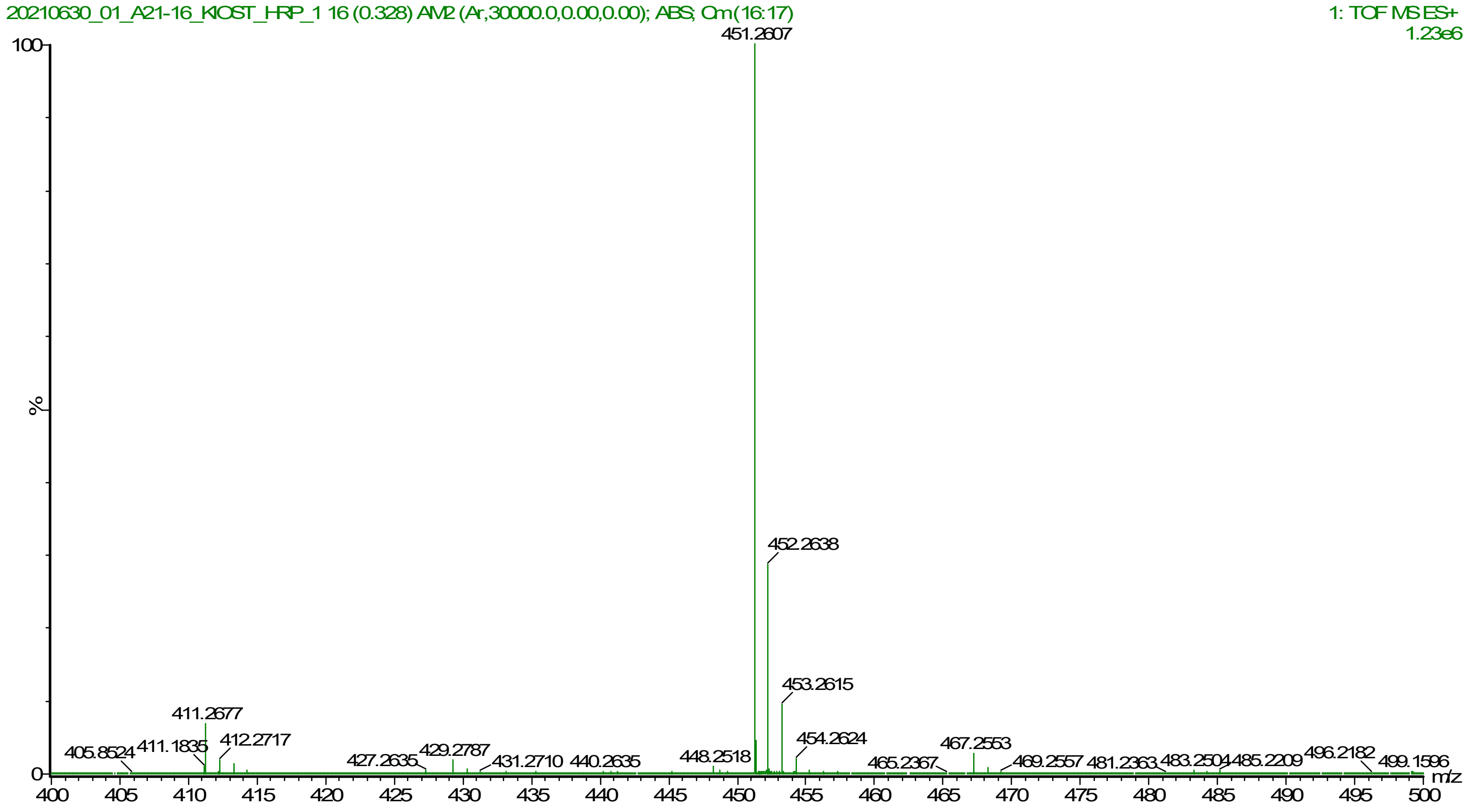

Minimum:				-5.0				
Maximum:		300.0	5.0	200.0				
Mass	Calc. Mass	mDa	PPM	DBE	i-FIT	Norm	Conf(%)	Formula
451.2607	451.2606	0.1	0.2	3.5	939.0	n/a	n/a	C22 H40 N2 O4 Na S

Figure S15. HR-ESIMS data of **2**.

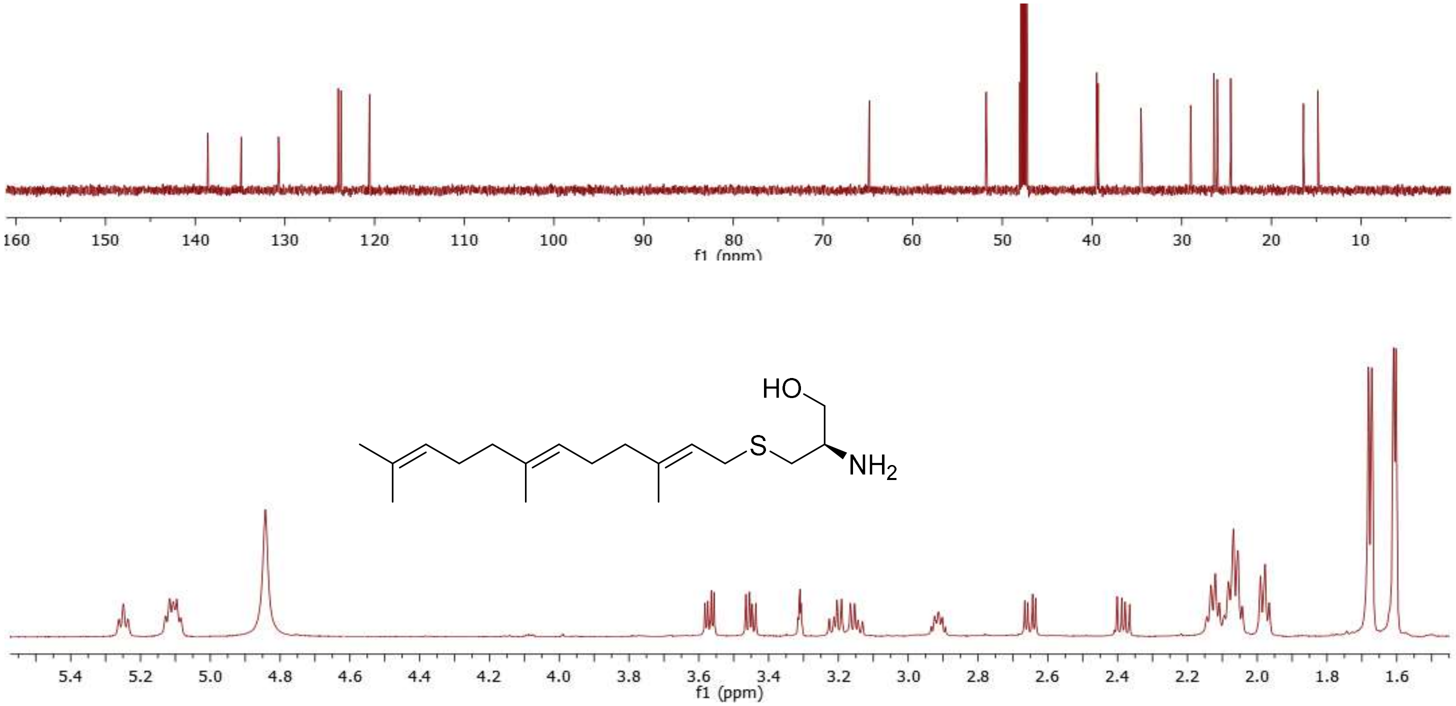

Figure S16. ^{1}H and ^{13}C NMR spectra of **6**.

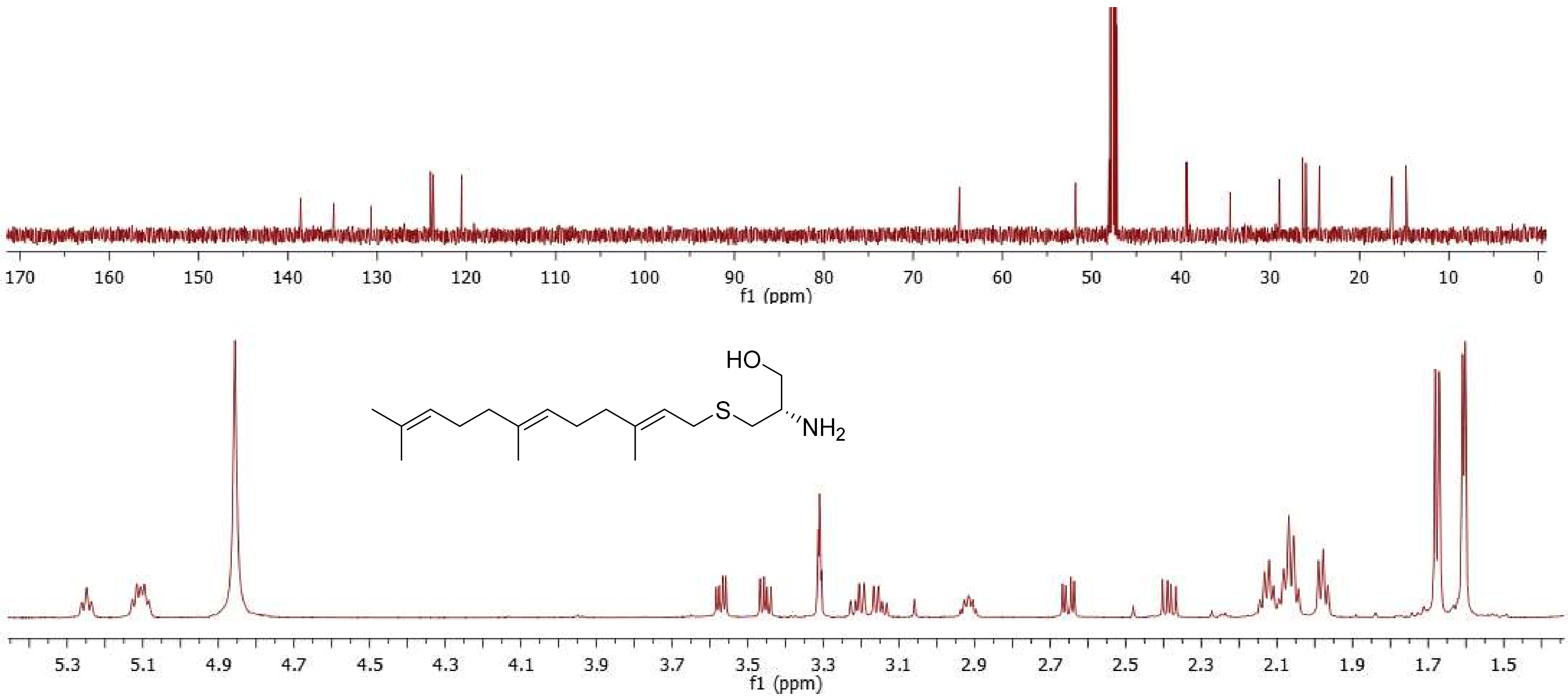

Figure S17. ^{1}H and ^{13}C NMR spectra of **7**.

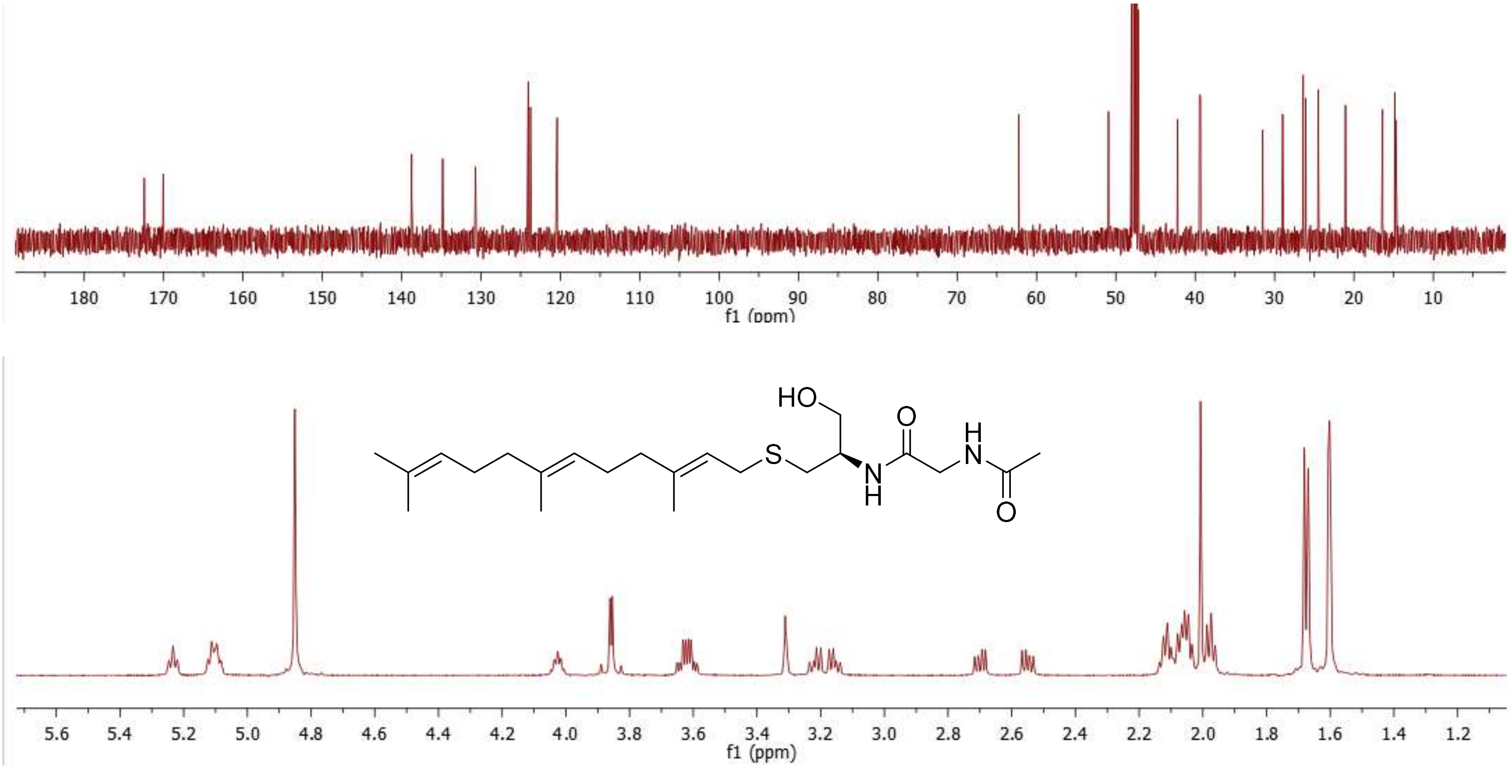

Figure S18. ^{1}H and ^{13}C NMR spectra of **4**.

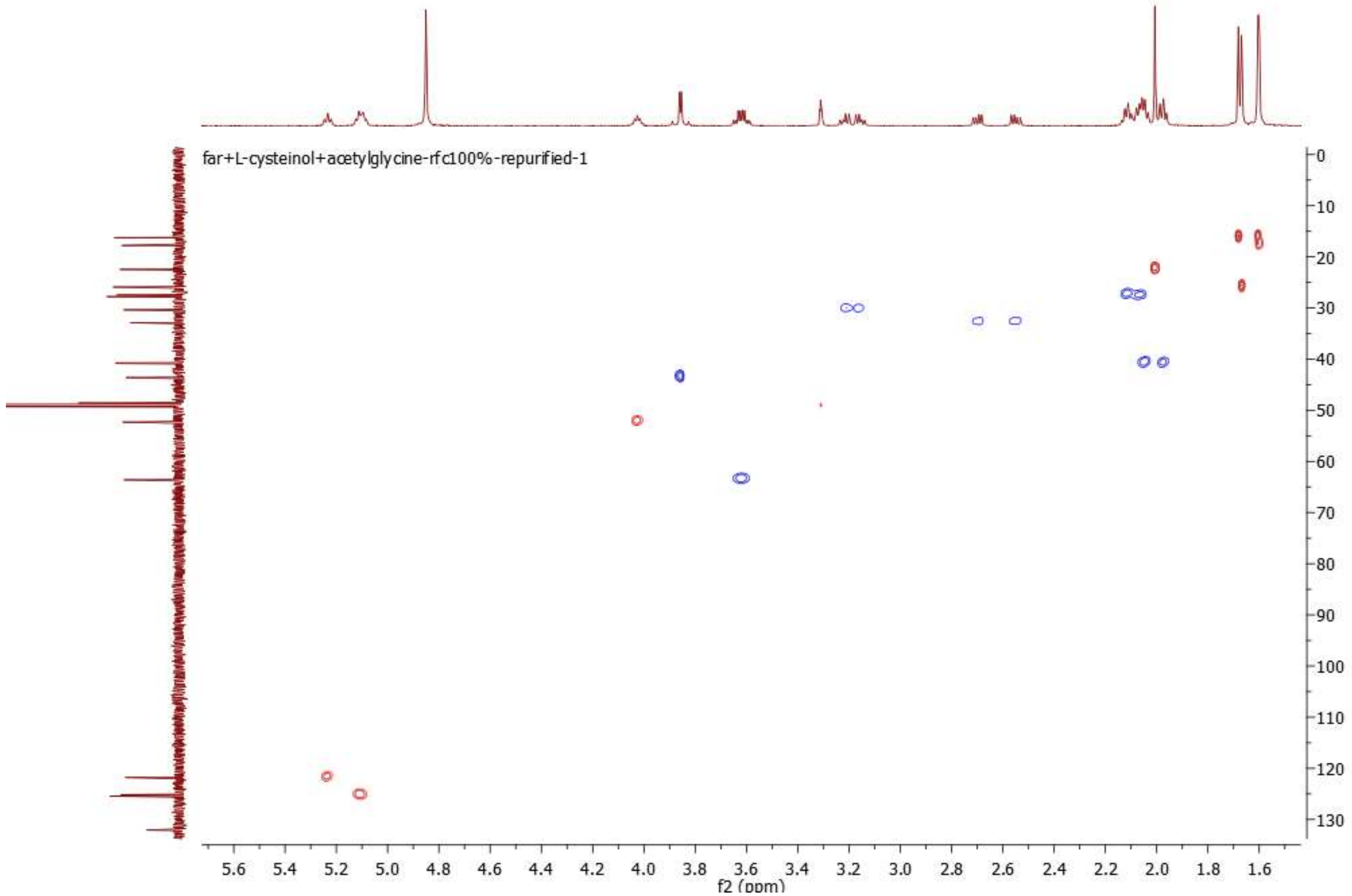

Figure S19. HSQC spectrum of **4**.

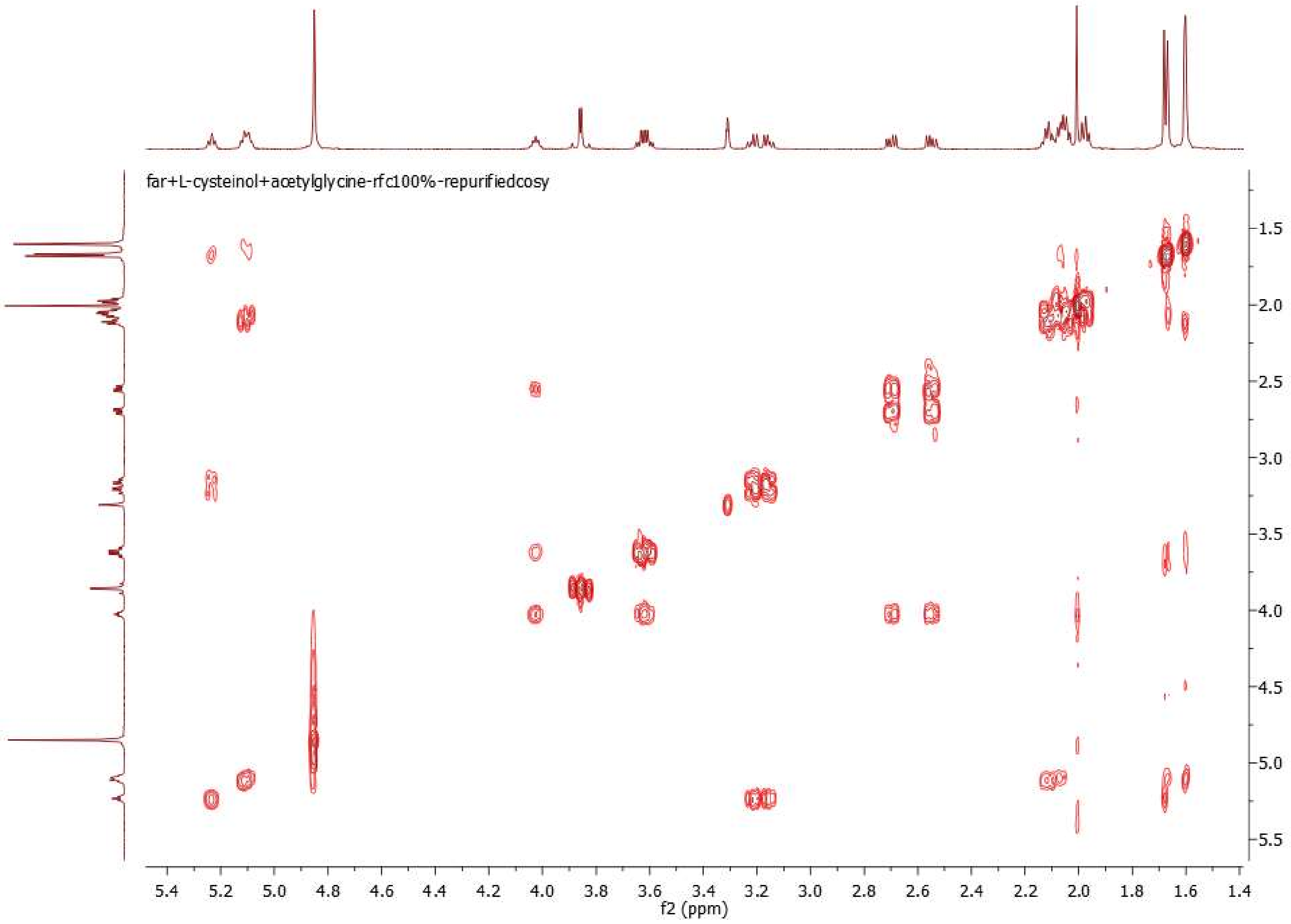

Figure S20. ^{1}H-^{1}H COSY spectrum of **4**.

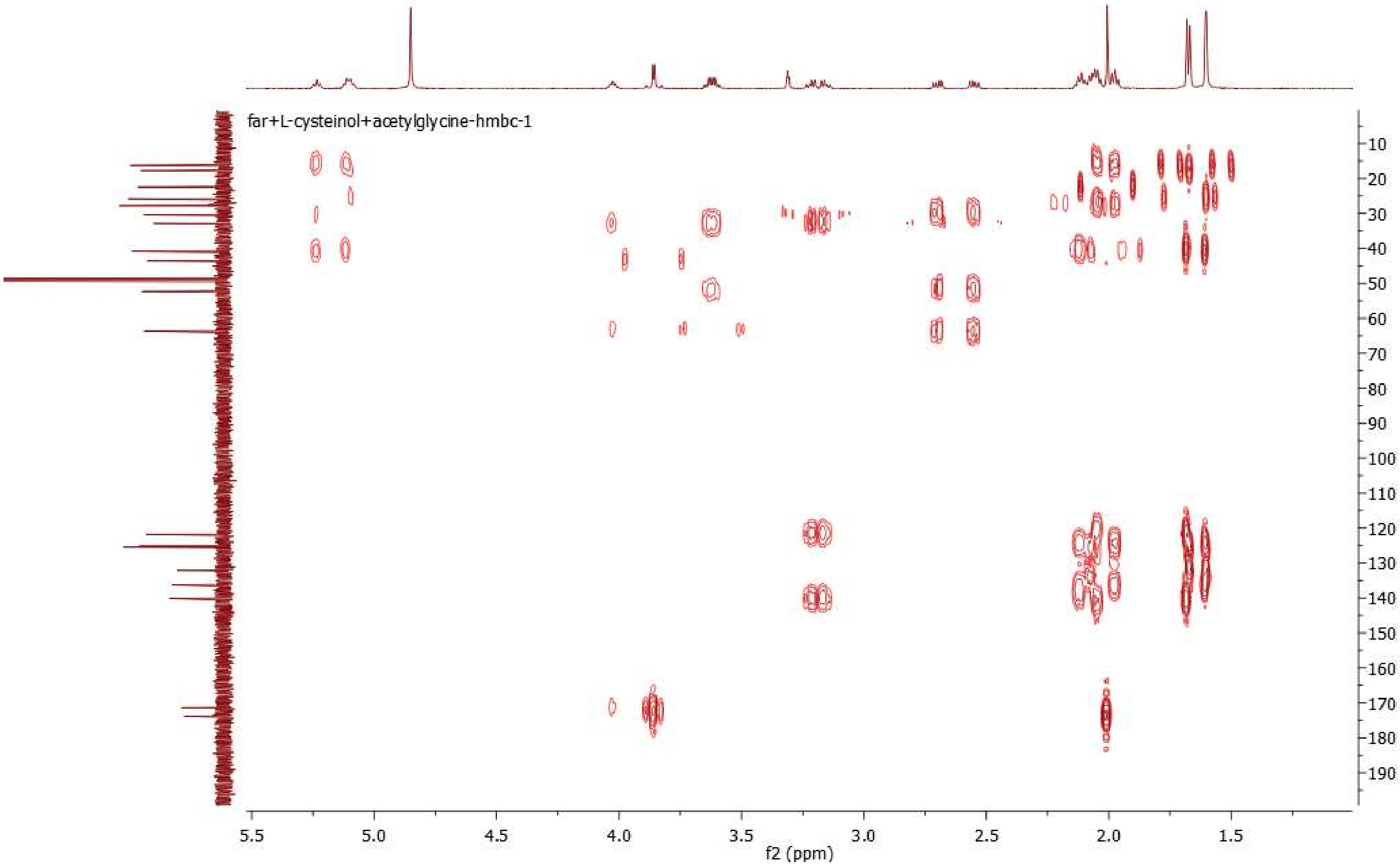

Figure S21. HMBC spectrum of **4**.

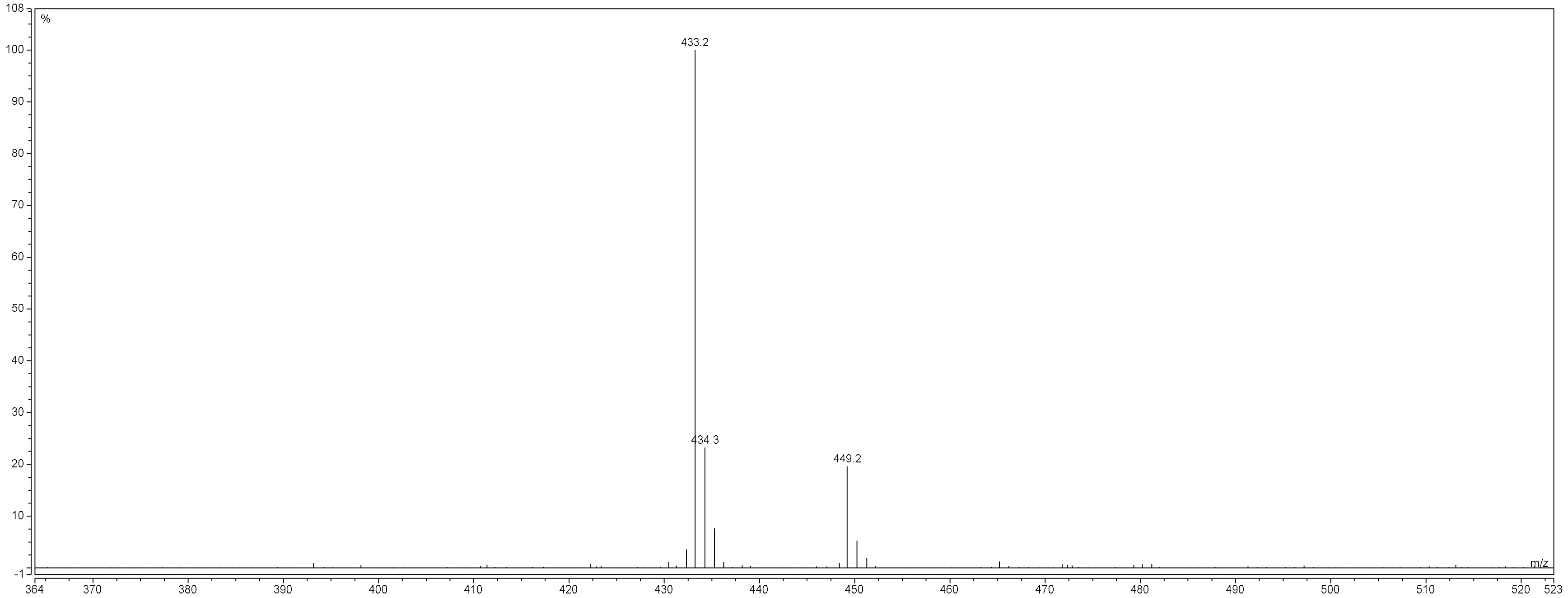

Figure S22. LC-ESIMS data of **4**.

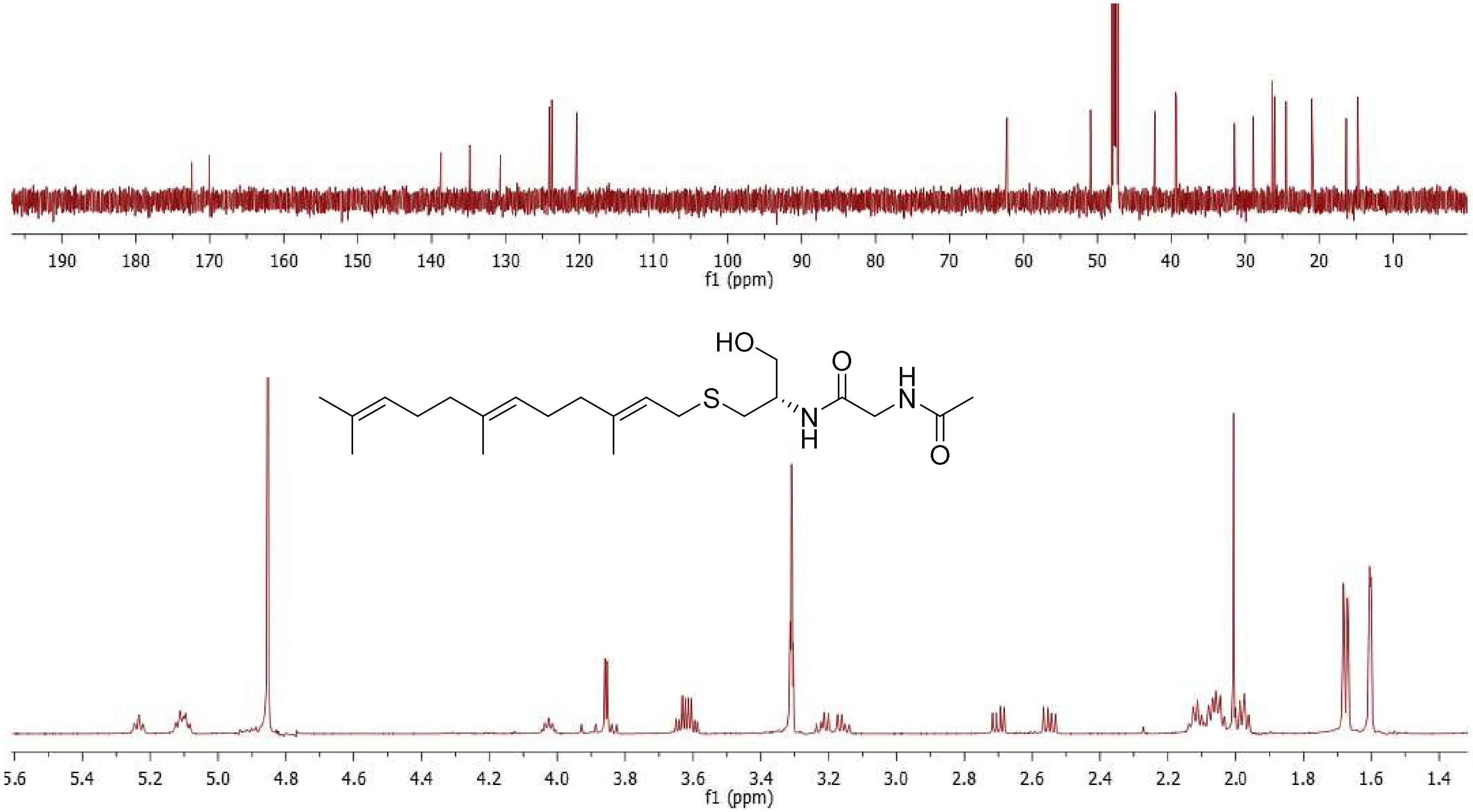

Figure S23. ^{1}H and ^{13}C NMR spectra of **5**.

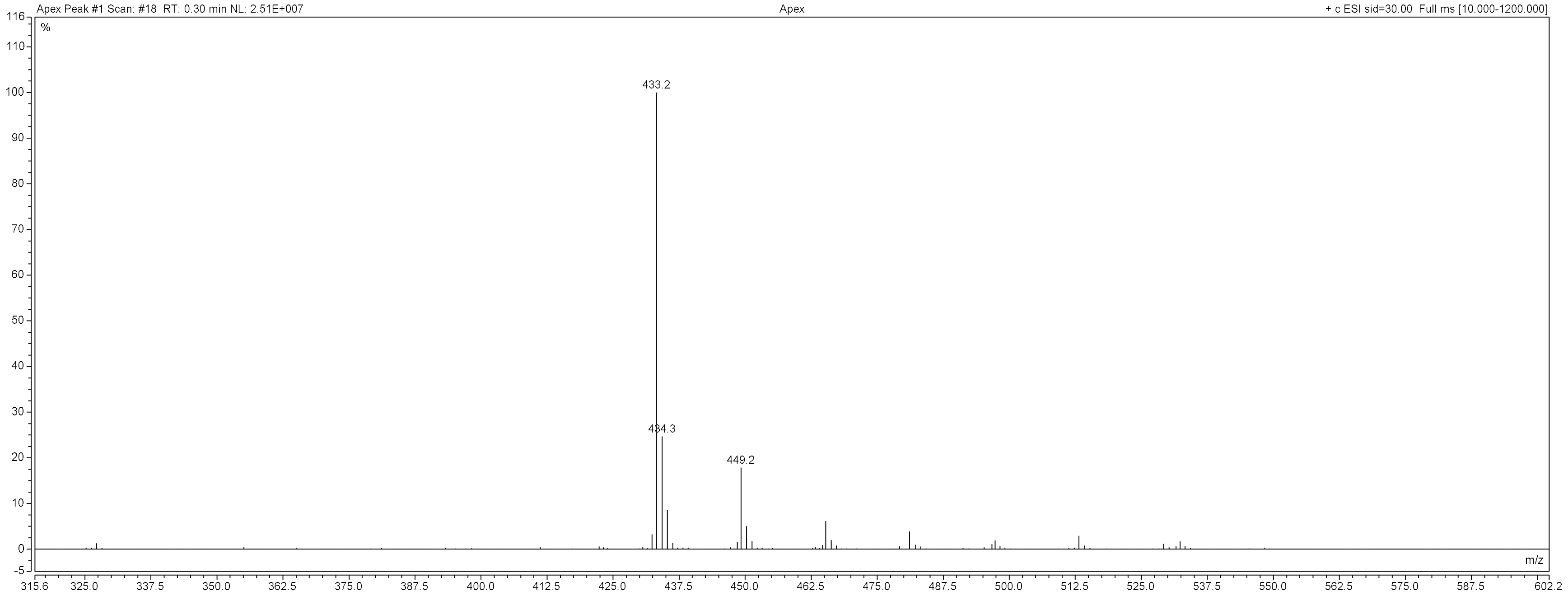

Figure S24. LC-ESIMS data of **5**.
